# Global, regional, and national time trends in disability-adjusted life years for neonatal preterm birth, from 1992 to 2021: an age-period-cohort analysis for the global burden of disease study 2021

**DOI:** 10.3389/fpubh.2025.1618151

**Published:** 2025-09-10

**Authors:** Yuhang Wu, Jianzhong Zuo, Chu Liu, Ziye Li, Le Zhang, Yao Zou, Xiaochan Wang, Lizhang Chen, Tingting Wang

**Affiliations:** ^1^Department of Epidemiology and Health Statistics, Xiangya School of Public Health, Central South University, Changsha, China; ^2^Department of Obstetrics, Xiangtan Central Hospital, Xiangtan, China; ^3^Hunan Provincial Key Laboratory of Clinical Epidemiology, Xiangya School of Public Health, Central South University, Changsha, China; ^4^NHC Key Laboratory of Birth Defect for Research and Prevention, Hunan Provincial Maternal and Child Health Care Hospital, Changsha, China

**Keywords:** age-period-cohort, disability-adjusted life years, global burden of disease, neonatal preterm birth, trends

## Abstract

**Background:**

Neonatal preterm birth emerges as a leading cause of neonatal mortality and morbidity. Although there is a growing recognition of the urgent need to address the global health challenges posed by neonatal preterm birth, the underlying causes behind the complex and multidimensional trends contributing to its burden, as well as potential intervention pathways, remain unclear. We aim to characterize and deeply analyze the global, regional, and national neonatal preterm birth burden and their trends from 1992 to 2021.

**Methods:**

Data on the number, all-age rate, age-standardized rate (ASR), and the relative change of neonatal preterm birth disability-adjusted life years (DALYs) were obtained from the Global Burden of Disease Study (GBD) 2021. Correlations of ASR with Socio-demographic Index (SDI) were evaluated by Spearman’s rank correlation analyses. Furthermore, age-period-cohort modeling was used to estimate the net drift (overall annual percentage change), local drift (age-specific annual percentage change), age, period, and cohort effects over the past three decades.

**Results:**

Globally, the number of neonatal preterm birth DALYs decreased by 34.0% from 121,633 in 1992 to 80,335 in 2021, and the ASR in 2021 was 1254.24 per 100,000 population, representing a reduction of 35.2% from 1992. The net drift ranged from 0.257% for high SDI region to 1.382% for low SDI region. A negative correlation was observed between ASR and SDI in 2021 at national levels. There has been a transition of DALYs from the neonatal population to older age groups (≥5 years) from 1992 to 2021. Regions and countries exhibited similar age-effect patterns, with decreasing risk with increasing age, and varying period and cohort effects.

**Conclusion:**

The global burden of neonatal preterm birth showed an overall declining trend from 1992 to 2021, but persistent health inequalities between regions and countries were driven by socioeconomic disparities. Neonatal preterm birth remains a crucial issue in children, and its growing long-term impact must not be overlooked. Each country presents unique trends, and tailored public health strategies in different settings are critical to managing the burden of preterm neonatal birth.

## 1 Introduction

Neonatal preterm birth, defined as delivery occurring before 37 completed weeks of gestation, constitutes a significant global public health challenge. Affecting approximately 15 million infants each year and accounting for 11% of all live births worldwide, preterm birth emerges as a leading cause of neonatal mortality and morbidity (1). It is associated with severe complications during the early stages of life, including respiratory distress syndrome, intraventricular hemorrhage, and necrotizing enterocolitis (2–4). Infants who survive preterm birth are at an increased risk for long-term health issues, which encompass neurodevelopmental disabilities, chronic health conditions, and impaired cognitive and developmental outcomes (5–7). The ramifications of preterm birth are considerable, resulting in substantial healthcare costs and social challenges for families and healthcare systems globally (8). Therefore, accurately assessing the disease burden associated with neonatal preterm birth is crucial for informing healthcare resource allocation and policy development aimed at alleviation.

Previous studies have reported the overwhelming burden of preterm births on a global scale, underscoring the urgency of timely attention and dedicated efforts to address this worldwide challenge (9, 10). However, these traditional epidemiological studies rely primarily on descriptive methods and fail to capture and reveal comprehensively the fundamental causes behind the trends in the complex, multidimensional factors contributing to the preterm birth burden and potential intervention pathways. The Age-Period-Cohort (APC) model provides a robust statistical framework for analyzing how age, period, and cohort effects affect the population burden of preterm birth, and it serves as a valuable tool for comprehensively investigating the epidemiological patterns of neonatal preterm births. With the release of the updated Global Burden of Diseases (GBD) 2021 database (11–13), there is a timely opportunity to examine the evolving trends and regional disparities related to neonatal preterm birth. The data from GBD 2021 can play a pivotal role in tracking the epidemiological dynamics within specific countries and offer essential evidence for developing targeted interventions, programs, and policies to improve health outcomes related to neonatal preterm birth.

Therefore, this study aims to quantify and characterize the global, regional, and national disability-adjusted life years (DALYs) attributable to neonatal preterm birth and their trends from 1992 to 2021. Additionally, the study employs the APC model to investigate the impacts of age, period, and cohort on the burden of neonatal preterm birth, offering valuable insights into the changing disease patterns. The findings are expected to guide public health strategies and inform policy initiatives aimed at mitigating the adverse outcomes associated with neonatal preterm birth, thereby highlighting its significance in advancing global health priorities.

## 2 Methods

### 2.1 Data source

This study utilized the GBD 2021 public dataset, available through the Global Health Data Exchange GBD Results Tool^1^. The GBD 2021 dataset offers detailed insights into the burden of 371 health conditions across 204 countries and territories, incorporating the latest epidemiological data and refined standardized methodologies (11–13). It also accounts for the impact of the COVID-19 pandemic on global disease burden estimates. In the GBD 2021, neonatal preterm birth is defined according to the International Classification of Diseases, 9th edition (ICD-9): 765.21–765.9, 769–770, 770.2–770.9, 776.6, 777.5–777.53 and 10th edition (ICD-10): P07.2-P07.39, P22-P22.9, P25-P28.9, P61.2, P77-P77.9 (11).

In this study, we obtained data on the number, all-age rates, and age-standardized rates (ASR) of DALY due to neonatal preterm birth, disaggregated by sex, region, and country, covering various age groups from 1992 to 2021. DALYs are calculated to assess the overall burden of disease by combining the years of life lost due to premature mortality with the years lived with disability, providing a comprehensive measure of the impact on public health. To account for variability in the data, 95% uncertainty intervals (UIs) were calculated by resampling the data 1,000 times, using the 2.5th and 97.5th percentiles to define the interval boundaries. The GBD 2021 also updated and produced a Socio-demographic Index (SDI) for 204 countries and territories, categorizing development status into five quintiles (Supplementary Table S1): high SDI (>0.81), high-middle SDI (0.70–0.81), middle SDI (0.61–0.69), low-middle SDI (0.46–0.60), and low SDI (<0.46). Detailed methodologies and the modeling approach for GBD 2021 have been documented in separate publications (11–13). The dataset used was anonymized and is publicly accessible, with the Institutional Review Board at the University of Washington approving a waiver of informed consent.

### 2.2 Data analysis

#### 2.2.1 Analysis of overall temporal trends in neonatal preterm birth DALYs

This study analyzed neonatal preterm birth burden and its spatial and temporal trends from 1992 to 2021. Temporal trends in DALYs over the study period were evaluated by DALYs number, all-age DALY rate (crude DALY rate) and ASR of DALY, and the relative change in percentage between 1992 and 2021. We then assessed the relationship between SDI values of the 204 countries and the ASR of DALYs (for both sexes) in 2021 by using Spearman’s rank correlation coefficient (rho). Furthermore, we examined the age distribution of neonatal preterm birth burden by categorizing number of DALYs into five age strata (0–4, 5–19, 20–39, 40–64, and 65–94 years) and calculating the proportion of DALYs in each age stratum.

#### 2.2.2 Age period cohort modeling analysis

The APC model is a statistical tool based on the Poisson distribution, which is used to extract and reveal potential information about disease trends (14). In this study, we applied the APC model to decompose the DALYs into three dimensions (age, period, and birth cohort), and analyze the corresponding effects on neonatal preterm birth DALYs. In the APC model, age effects represent the different risks of the outcome associated with different age brackets; the period effects capture changes in the outcome over time that affect all age groups simultaneously; and the cohort effects reflect changes in the outcome across groups of people who share the same birth year (15). The APC model can be formulated as follows:


$$g(Y_{j}/\mu )=\log(\lambda_{j})=u+\alpha \;{\mathrm{age}}_{j}+\beta \;{\text{period}}_{j}+\gamma \;{\text{cohort}}_{j}$$

where $\lambda_{j}$ represents the response variable of the net effect on neonatal preterm birth DALYs rate for group j ; $Y_{j}$ and μ represent the number of DALYs and the population at risk, respectively. α , β , and γ represent the coefficients of age, period, and birth cohort of the APC model, respectively. u represents the intercept of the model.

The GBD 2021 DALYs estimates for neonatal preterm birth and population data of each country or region were used as inputs for the APC model with the intrinsic estimator (IE) method. The IE method, applied in this study, addresses the issue of parameter indeterminacy inherent in the APC model’s age, period, and cohort effects. More methodological information is available in the previous literature (16). In this model, equal intervals for age and period are required; therefore, the population aged 0–94 years was divided into 19 age groups (0–4, 5–9, …, 90–94) in successive 5-year intervals. To account for short-term fluctuations, such as those from the COVID-19 pandemic, the GBD data were incorporated into a single unit framework by selecting the DALYs and population counts from the mid-year of six time point values (1994, 1999, …, 2019) rather than the 5-year averages to represent the specific period, although the data provided by these two processing methods have been found to be very similar in a previous study (17). The input data included 19 age groups and 21 consecutive cohorts, defined by mid-year birth intervals from 1900 to 1904 (median 1902) to 2015–2019 (median 2017).

In this study, we primarily focus on the following estimable functions. The net drift represents the overall annual percentage change for DALY rates over time. Local drifts reflect annual percentage changes by period and cohort for each age group, while the longitudinal age curve indicates the fitted longitudinal age-specific rates adjusted for period deviations within the reference cohort. The period (or cohort) rate ratio (RR) refers to the ratio of age-specific rates in each period (or cohort) relative to the reference one. APC analyses were conducted using the National Cancer Institute’s age-period-cohort web-based tool, with subsequent data visualization and statistical analysis performed in R (version 4.2.3). Statistical significance for parameters was assessed using the Wald χ^2^ test, with all tests being two-tailed.

## 3 Results

### 3.1 Global, regional and countries trends in neonatal preterm birth DALYs, 1992–2021

From 1992 to 2021, the total number of DALYs attributed to neonatal preterm birth was 80,335 thousand (95% UI: 69876 to 93,717) in 2021, reflecting a decrease of 34.0% compared to 1992. The global ASR of neonatal preterm birth DALYs in 2021 was 1254.24 (95% UI: 1088.18 to 1465.54) per 100,000 population, representing a reduction of 35.2% from 1992. The APC model estimated a net drift of 1.165% (95% confidence interval [CI] 0.597 to 1.735) in the neonatal preterm birth DALY rates globally from 1992 to 2021 (Table 1).

**Table 1:** Trends in neonatal preterm birth disability-adjusted life years across Socio-demographic Index quintiles, 1992–2021.

Characteristic	Global (*N* = 204)		High SDI (*N* = 40)		High-middle SDI (*N* = 47)		Middle SDI (*N* = 41)		Low-middle SDI (*N* = 43)		Low SDI (*N* = 33)	
	1992	2021	1992	2021	1992	2021	1992	2021	1992	2021	1992	2021
Population												
Number, n x 1,000,000	5,497 (5,379, 5,624)	7,891 (7,667, 8,131)	894 (868, 919)	1,094 (1,064, 1,125)	1,087 (1,045, 1,131)	1,304 (1,251, 1,360)	1780 (1717, 1839)	2,449 (2,354, 2,542)	1,206 (1,164, 1,247)	1921 (1821, 2023)	526 (513, 540)	1,117 (1,068, 1,166)
Percentage of global, %	100	100	16.3	13.9	19.8	16.5	32.4	31.0	21.9	24.3	9.6	14.2
DALYs												
Number, n x 1,000	121,633 (112,203, 130,772)	80,335 (69,876, 93,717)	3,888 (3,604, 4,232)	2,216 (1884, 2,557)	9,416 (8,631, 10,453)	2,781 (2,407, 3,171)	32,587 (30,233, 35,399)	14,941 (12,931, 17,074)	50,629 (45,731, 55,674)	32,461 (27,390, 38,092)	25,040 (22,445, 27,752)	27,888 (23,626, 33,171)
Percentage of global, %	100	100	3.2	2.8	7.7	3.5	26.8	18.6	41.6	40.4	20.6	34.7
Percent change of DALYs 1992–2021, %	−34.0		−43.0		−70.5		−54.2		−35.9		11.4	
All-age DALYs rate												
Rate per 100,000	2212.63 (2041.08, 2378.88)	1018.02 (885.47, 1187.59)	435.11 (403.30, 473.63)	202.61 (172.16, 233.72)	866.61 (794.40, 962.05)	213.25 (184.57, 243.19)	1830.94 (1698.70, 1988.98)	610.19 (528.13, 697.30)	4198.72 (3792.51, 4617.09)	1689.70 (1425.75, 1982.80)	4757.09 (4264.23, 5272.34)	2495.85 (2114.38, 2968.64)
Percent change of rate 1992–2021, %	−54.0		−53.4		−75.4		−66.7		−59.8		−47.5	
Age-standardized DALYs rate												
Rate per 100,000	1936.42 (1787.57, 2083.71)	1254.24 (1088.18, 1465.54)	609.68 (572.00, 653.26)	334.94 (296.63, 374.55)	1125.83 (1030.76, 1247.31)	383.41 (342.02, 431.67)	1675.97 (1554.65, 1820.55)	887.08 (763.42, 1022.90)	2764.56 (2502.54, 3039.54)	1718.94 (1449.88, 2019.09)	2348.79 (2114.52, 2594.98)	1667.11 (1421.01, 1982.42)
Percent change of rate 1992–2021, %	−35.2		−45.1		−65.9		−47.1		−37.8		−29.0	
APC model estimates												
Net drift of DALYs rate, % per year	1.165 (0.597, 1.735)		0.257 (−0.044, 0.558)		0.589 (−0.008, 1.189)		1.372 (0.517, 2.233)		1.189 (0.350, 2.035)		1.382 (0.523, 2.245)	

All-age DALYs rate: crude DALYs rate.

Age-standardized DALYs rate is computed by direct standardization with global standard population in the GBD 2021.

Net drifts are estimates derived from the age-period-cohort model and denotes overall annual percentage change in DALYs rate, which captures the contribution of the effects from calendar time and successive birth cohorts.

DALYs, disability-adjusted life years, APC: age-period-cohort, UI: uncertainty interval, CI: confidential interval.

Regionally, the ASR of neonatal preterm birth DALYs was highest in the low-middle SDI (1718.94 per 100,000 population, 95% UI: 1449.88 to 2019.09) and low SDI regions (1667.11 per 100,000 population, 95% UI: 1421.01 to 1982.42), while it was lower in the high SDI (334.94 per 100,000 population, 95% UI: 296.63 to 374.55) and high-middle SDI (383.41 per 100,000 population, 95% UI: 342.02 to 431.67) regions in 2021. Notably, the annual net drift of DALY rates ranged from 0.257% (95% CI: −0.044 to 0.558) for high SDI region to 1.382% (95% CI: 0.523, 2.245) for low SDI region (Table 1).

**Figure 1:**
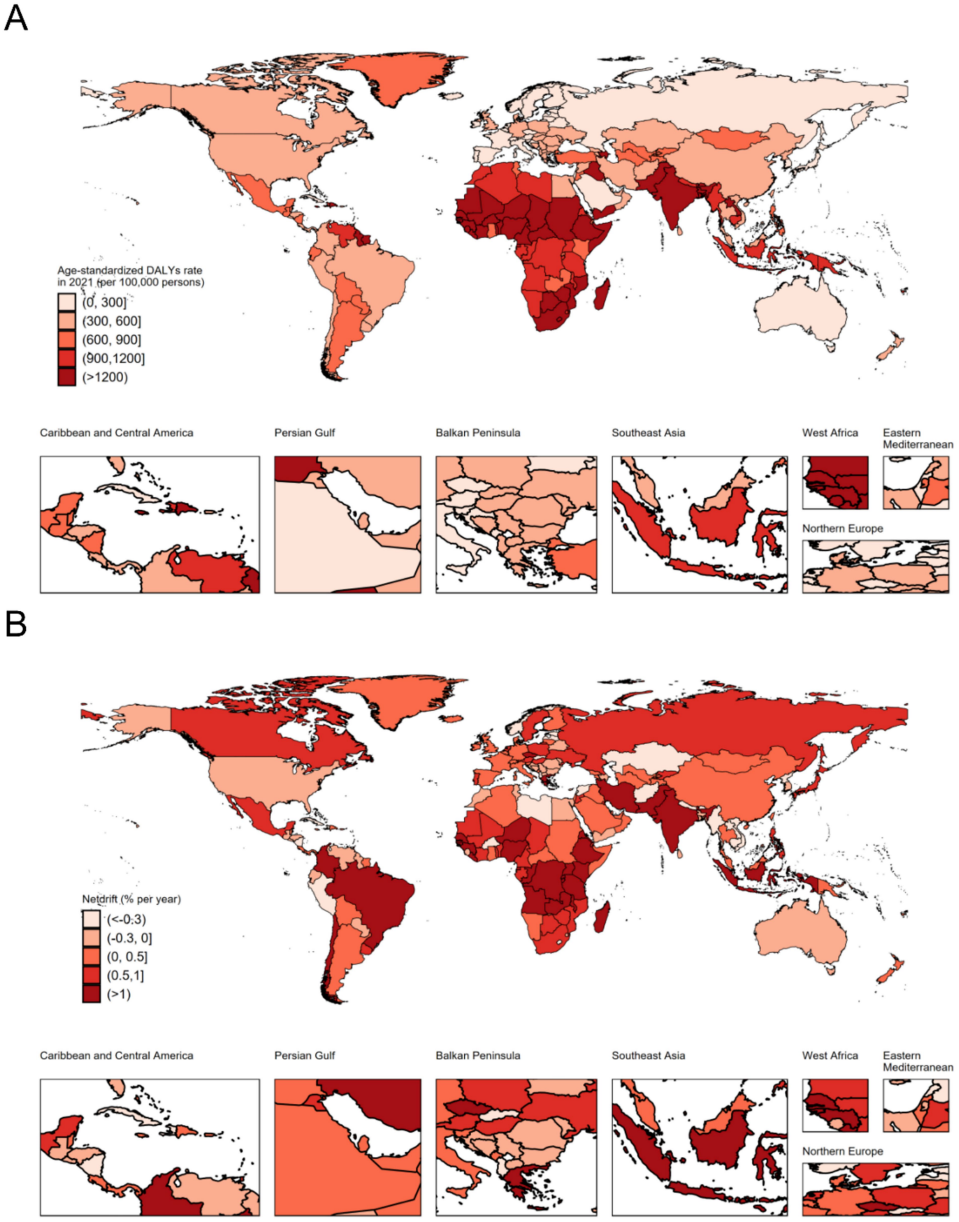
The age-standardized disability-adjusted life years rate in 2021 **(A)**, and net drift of disability-adjusted life years rate during 1992–2021 **(B)** for neonatal preterm birth in 204 countries and territories. Net drift captures components of the trends attributable to calendar time and successive birth cohorts. DALYs, disability-adjusted life years.

**Figure 2:**
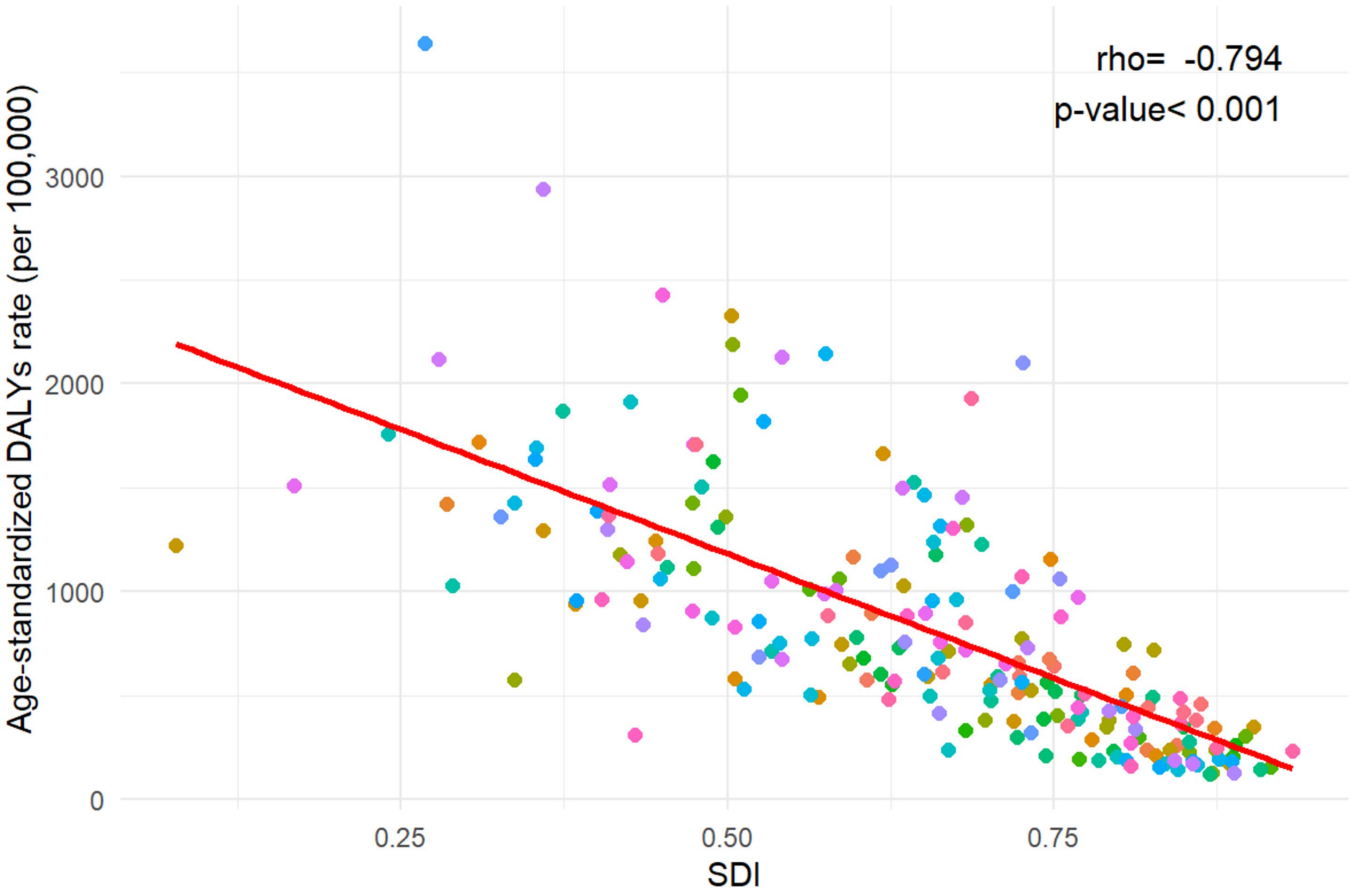
The association between age-standardized disability-adjusted life years rate for neonatal preterm birth and Socio-demographic Index in 204 countries and territories. DALYs, disability-adjusted life years; SDI, Socio-demographic Index.

At the national level, India had the highest number of DALYs at 24,143 thousand (95% UI: 19,826 to 29,610), followed by Nigeria (9,031 thousand, 95% UI: 6,955 to 11,466), Pakistan (6,415 thousand, 95% UI: 4,957 to 8,258), and China (2,114 thousand, 95% UI: 1,782 to 2,467). These four countries accounted for more than half of the global burden in 2021. The highest ASIR was observed in Mali (3640.04 per 100,000 population, 95% UI: 2940.35 to 4462.02), and the lowest was recorded in Andorra (124.02 per 100,000 population, 95% UI: 91.59 to 160.11). More than 90% of the countries experienced a decrease in ASR of DALYs from 1992 to 2021. The annual net drift of DALYs ranged from-1.708% (95% CI: −2.005 to-1.410) in the Democratic People’s Republic of Korea to 3.307% (95% CI: −0.353 to 7.101) in Ethiopia (Supplementary Table S2, Figure 1). Furthermore, a significant negative correlation was observed between ASR of neonatal preterm birth DALYs and SDI values in 2021 (rho = −0.794; *p* < 0.001) (Figure 2).

### 3.2 Time trends in neonatal preterm birth DALY rate across different age groups

**Figure 3:**
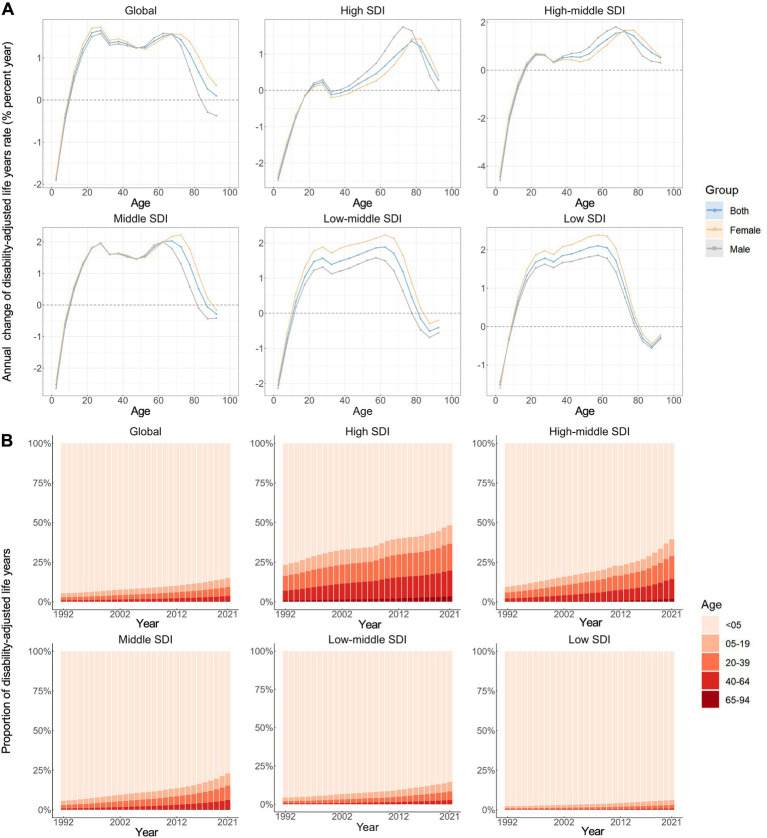
Local drifts of age-standardized disability-adjusted life years rate and age distribution of disability-adjusted life years by SDI quintiles, 1992–2021. **(A)** Local drifts of neonatal preterm birth disability-adjusted life years rate (estimates from age-period-cohort models) for 19 age groups (0–4 to 90–94 years), 1992–2021. The dots and shaded areas indicate the annual percentage change of disability-adjusted life years rate (% per year) and the corresponding 95% CIs. **(B)** Temporal change in the relative proportion of neonatal preterm birth disability-adjusted life years across age groups (<5, 5–19, 20–39, 40–64, 65–94 years), 1992–2021. SDI, Socio-demographic Index.

Figure 3A illustrates the annual percentage change in the DALY rates of neonatal preterm birth for each 5-year age group ranging from 0 to 94 years. Specifically speaking, only the young age groups (0–4 and 5–9 years) demonstrated predominantly negative local drift values, while positive local drift values were shown in the other age groups (10–94 years), globally. A similar trend was observed among five SDI regions. Noteworthy, negative local drift values were also observed for older age groups (85–89 and 90–94 years) in middle, low-middle and low SDI regions. Moreover, the local drift of DALY rates for each country is shown in Supplementary Figures S1–S5.

Figure 3B illustrates the temporal trends in the number of disability-adjusted life years (DALYs) attributed to neonatal preterm birth by age group. Overall, the majority of DALYs was recorded among children under 5 years of age (0–4 years), with similar distributions observed across all SDI regions. Concurrently, there has been a noteworthy transition of DALYs from the neonatal population to older age groups (≥5 years) from 1992 to 2021, a trend that is more pronounced in regions with higher SDI. The age distribution of DALYs for each country is presented in Supplementary Figures S6–S10.

### 3.3 Age, period, and cohort effects on neonatal preterm birth DALY rate

**Figure 4:**
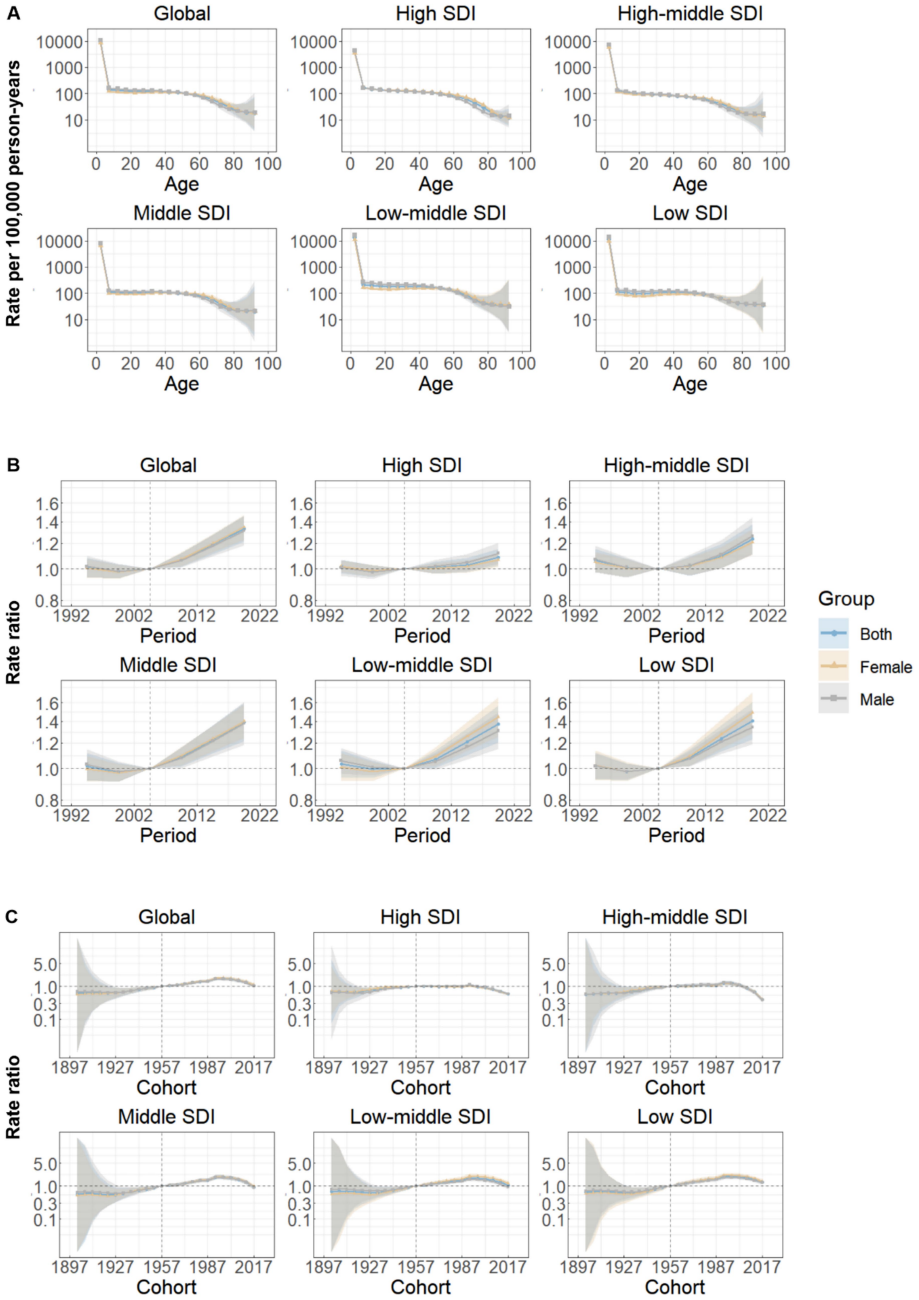
Age, period and cohort effects on neonatal preterm birth disability-adjusted life years rate by SDI quintiles. **(A)** Age effects are shown by the fitted longitudinal age curves of disability-adjusted life years rate (per 100,000 person-years) adjusted for period deviations. **(B)** Period effects are shown by the relative risk of disability-adjusted life years rate (rate ratio) and computed as the ratio of age-specific rates from 1992–1996 to 2017–2021, with the referent cohort set at 2002–2006. **(C)** Cohort effects are shown by the relative risk of disability-adjusted life years rate and computed as the ratio of age-specific rates from the 1902 cohort to the 2017 cohort, with the referent cohort set at 1957. The dots and shaded areas denote incidence rates or rate ratios and their corresponding 95% CIs. SDI, Socio-demographic Index.

Figure 4 shows the estimates for age, period, and cohort effects for both global data and five SDI regions. Overall, a similar pattern of age effects was observed across all areas, with the rate of DALYs attributed to neonatal preterm birth demonstrating a downward trend with increasing age in the reference cohort after adjusting for period effects. Notably, both males and females aged 0–4 years exhibited the highest DALY rates (Figure 4A).

Period effects displayed a gradual increase globally over the past three decades, with analogous trends observed across the five SDI regions. Furthermore, the magnitude of period effects was comparatively smaller in regions with higher SDI (Figure 4B).

Globally, cohort effect exhibited a slight increase among successive birth cohorts prior to the 21st century, followed by a declining trend in birth cohorts after 2002. This pattern was particularly evident in low, low-middle, and middle SDI regions. However, in high and high-middle SDI regions, the risk ratio of birth cohort initially increased, followed by a cohort of relative stability, and subsequently began to decline (Figure 4C). The age, period, and cohort effects on neonatal preterm birth DALY rates in each country are detailed in Supplementary Figures S11–S25.

### 3.4 Age-period-cohort effects in representative countries

In this study, we present representative countries from various Sociodemographic Index (SDI) regions to more accurately illustrate the trends in disease burden associated with neonatal preterm birth. Supplementary Figure S26 displays countries exhibiting favorable age-period-cohort effects. As a representative nation of the high SDI regions, the United States of America demonstrated a general slow decline in both period and cohort effects. Additionally, the emerging age distribution indicates that the burden of neonatal preterm birth is gradually affecting all age groups. Belarus recorded the most significant decline in disability-adjusted life years (DALYs) among high-middle SDI regions over the past three decades, with a reduction of 79.70%. Both Cuba and Nepal showed a decline in DALY rates across all age groups (with local drifts < 0), accompanied by a gradual decrease in relative risk over time and across birth cohorts. However, Cuba has exhibited a slight upward trend in recent years. Democratic People’s Republic of Korea stood out due to its significant net drift (−1.708%) and exhibited steadily declining period and cohort effects.

Supplementary Figure S27 presents countries exhibiting unfavorable age-period-cohort effects. Driven by increasing period and cohort effects, Kuwait experienced one of the smallest declines in DALY rates, with a change of 17.34%, among high SDI countries from 1992 to 2021, as the total number of DALYs increased by 45.18%. Both Chile and Brazil, located in South America, demonstrated positive local drift values across nearly all age groups (with the exception of ages 0–10), characterized by relatively similar period and cohort effects, and exhibiting progressively worsening period risks and elevated birth cohort risks prior to the 2000s. In 2021, India contributed nearly one-third of global DALYs. However, despite a decline in ASR of DALY over the past three decades, the associated risk continued to rise over time. Ethiopia showed the most adverse trends in DALY rates related to neonatal preterm birth among 204 countries and territories, after controlling for age, period, and cohort effects, with a notable net drift of 3.307%. Furthermore, similar to other low-SDI countries, the risk significantly increased across the entire period and among individuals born before 2000.

## 4 Discussion

This study presents a detailed examination of the global burden associated with neonatal preterm birth from 1992 to 2021, highlighting an overall decreasing trend in DALYs during this period. Our analysis identified a significant negative correlation between the SDI and ASR of DALY, indicating that regions with lower SDI are disproportionately affected by this health issue. While neonatal preterm birth DALYs continue to be predominantly attributed to the neonatal age group, there has been an observed transition in the age distribution of DALYs toward higher age groups over the observation period. Additionally, the APC model underscored the complexity of the epidemiology of neonatal preterm birth, reflecting profound health disparities at global, regional, and national levels in terms of age, period, and cohort effects. These findings highlight the need for targeted interventions and policies that respond to the unique burden of neonatal preterm birth across different populations and contexts.

The results of our study indicate that both the absolute number and rate of DALYs due to neonatal preterm birth have generally followed a declining trend over the past few decades. However, the extent of this decline varies significantly across different regions and countries, with high SDI regions exhibiting more substantial reductions compared to lower SDI regions. These variations can be attributed to improvements in neonatal care, maternal health policies, and healthcare infrastructure in high SDI areas, where greater resources have been allocated to prevent neonatal preterm births and manage complications (18, 19). Conversely, in low SDI regions, challenges such as limited access to quality healthcare, inadequate neonatal facilities, and delayed implementation of effective interventions continue to impede progress (19, 20). The negative correlation observed in our analysis between SDI and ASR of DALY underscores the persistent disparities in neonatal health outcomes, particularly the disproportionate burden of neonatal preterm birth-related complications in lower SDI countries. Furthermore, our study found an apparent contradiction between the positive net drift values and the declining ASR of DALY from 1992 to 2021. A positive net drift indicates that the underlying health risks for neonatal preterm birth may have increased overall, potentially due to factors such as early maternal age, increasing rates of modern lifestyles that could impact maternal health, or heightened detectability of neonatal preterm births (21). The decline in ASR reflects effective healthcare interventions, improved neonatal care, and changes in the survival rates of preterm infants (22). This inconsistency implies that while advancements in neonatal care and health systems are yielding benefits, the fundamental risk factors contributing to neonatal preterm birth may still be growing in certain populations.

Significant disparities in the burden of neonatal preterm birth exist among countries, with biological, genetic, and socio-environmental factors influencing the complications associated with neonatal preterm birth in various contexts (23). Recent inquiries have raised questions about the relationships among these factors and neonatal preterm birth-related disease burden, underscoring the necessity of exploring age, period, and cohort effects to better understand these disparities. A consistent age effect pattern indicates that the most severe health impacts of neonatal preterm birth predominantly occur early in life, with a gradual reduction in burden as children grow older. This trend may be due to improved survival rates, better management of early-life complications in resource-rich settings, and advancements in neonatal care that have significantly enhanced outcomes for preterm infants (22, 24). Early intervention and comprehensive neonatal care need further strengthening to address the immediate health challenges faced by premature infants. Nevertheless, the residual effects of neonatal preterm birth, including potential long-term developmental and health issues (25), continue to impact disease burden throughout childhood and possibly into adulthood. This shift underscores the necessity of viewing neonatal preterm birth as a lifelong health issue. Effective long-term interventions are essential for mitigating chronic impacts and reducing the overall burden across the lifespan.

We further employed the APC framework to focus on patterns of neonatal preterm birth disease burden in countries represented in different SDI regions (Supplementary Figures S26, S27). In the United States, the gradual decline in both period and cohort effects likely reflects advancements in neonatal care and improved access to healthcare (18). However, the ongoing dissemination of disease burden across all age groups suggests that while acute neonatal care has improved, long-term developmental challenges persist (26), necessitating continued investment in early childhood interventions and sustained support for preterm survivors. In Belarus, the significant reduction in DALYs over the past three decades suggests the effectiveness of targeted maternal and child health policies. Comprehensive prenatal care, public health education, and advancements in neonatal technology have likely contributed to these positive outcomes (27). Efforts in Cuba and Nepal to expand access to skilled birth attendants, increased use of antenatal care, and community-based interventions aimed at reducing preterm births, especially in rural areas, were supported by international assistance and Government-led initiatives to address health disparities and improve maternal outcomes (28–30). Nevertheless, the recent slight increase in burden in Cuba may indicate limitations within its healthcare system under economic pressures (31). The health-care system of the Democratic People’s Republic of Korea provides free, State-controlled maternal and neonatal care (32), The highly regulated social structure and extensive public health education may have enforced compliance with healthcare recommendations. However, the closed nature of the country limits external validation of these results, and the lack of transparency may obscure other underlying factors contributing to these trends (33).

Kuwait faces significant challenges in addressing the risk factors and long-term impacts associated with neonatal preterm birth. Obesity, diabetes, and delayed childbearing are more prevalent in high SDI countries (21, 34, 35). Additionally, the implementation of comprehensive maternal and child health programs, which focus on both prevention and long-term management of preterm-related conditions, has been slower (36). In Chile and Brazil, persistent socioeconomic inequalities significantly impact preterm birth rates (37). Economic fluctuations in Chile have also worsened access to healthcare for pregnant women (38). Although the Family Health Strategy has improved healthcare access in some regions (39), poverty-stricken areas continue to experience high preterm birth rates due to geographic barriers and insufficient healthcare resources in Brazil (40). Although India has made strides in improving maternal and neonatal healthcare through initiatives like the National Urban Health Mission, further efforts are needed to close the healthcare disparity gap and mitigate rising risks over time (41). The alarming trend in Ethiopia illustrates a dilemma common to many low SDI countries, with limited healthcare infrastructure, high rates of malnutrition, and low access to prenatal care (42, 43). While international aid and local efforts have aimed to improve maternal and neonatal health, the example of Ethiopia shows a clear need for more comprehensive strategies to address the root causes of neonatal preterm birth and improve neonatal outcomes in underdeveloped regions.

We acknowledge the remarkable progress made globally in recent years toward reducing the disease burden associated with neonatal preterm birth, which is encouraging. However, several critical issues require continued attention. First and foremost, enhancing access to quality prenatal care is essential. Countries should invest in maternal health programs that offer comprehensive education and support for expectant mothers, with particular attention to those disadvantaged by social or geographical factors, regardless of whether their regions are classified as high or low SDI (44, 45). Community-based interventions can be particularly effective, as they raise awareness of preterm birth risk factors and incentivize early and consistent medical consultations (46). Meanwhile, the long-term health impacts of preterm birth necessitate an integrated approach that extends beyond the neonatal period. This includes strengthening follow-up care and support services for preterm infants, ensuring that survivors receive adequate healthcare throughout their lives to mitigate the risk of chronic conditions associated with preterm birth. Additionally, the substantial disparities in disease burden across regions and countries require governments to develop public health strategies that consider local socio-economic conditions and healthcare capacities. Such contextualized and evidence-based approaches will be essential for maximizing the effectiveness of these interventions.

Here, the detailed APC analysis effectively captures the independent effects of age, period, and cohort on trends in neonatal preterm birth burden at global, regional, and national levels, offering valuable guidance for future policy and clinical interventions. However, several limitations must be acknowledged. First, the data for GBD 2021 were derived from various sources, including surveys, registries, and administrative records, each differing in quality and completeness. This variability introduces potential bias and uncertainty into the conclusions. Second, the GBD database often relies on modeled estimates for regions with sparse direct data. The assumptions underlying these models may not be universally applicable, particularly considering the impact of diverse cultural, genetic, and environmental factors on neonatal preterm birth burden.

## 5 Conclusion

This study provides a comprehensive overview of the global burden of neonatal preterm birth from 1992 to 2021, revealing an overall decline in DALYs while highlighting persistent health inequalities across regions and countries driven by socioeconomic disparities. Neonatal preterm birth remains a crucial issue in children, and its growing long-term impact must not be overlooked. Moreover, our study underscores the complex interplay of age, period, and cohort effects on neonatal preterm birth. Each country exhibits unique trends shaped by its socio-economic, cultural, and historical contexts, indicating that tailored public health strategies are crucial for effectively addressing and managing the neonatal preterm birth burden in diverse settings. Future research should explore the impact of specific policies and interventions to reduce the burden of neonatal preterm birth across the lifespan.

## Data Availability

Publicly available datasets were analyzed in this study. This data can be found here: the datasets generated during and/or analyzed during the current study are available in the GBD Data Tool repository (http://ghdx.healthdata.org/gbd-results-tool). This public link to the database of GBD study is open, and the use of data does not require additional consent from IHME.

## Footnotes

^1^ https://ghdx.healthdata.org/gbd-2021
