# Supplementary material for: Global, regional, and national time trends in disability-adjusted life years for neonatal preterm birth, from 1992 to 2021: an age-period-cohort analysis for the global burden of disease study 2021
Source: Front Public Health. 2025 Sep 10;13:1618151. doi: 10.3389/fpubh.2025.1618151 (PMC12457167; doi:10.3389/fpubh.2025.1618151)
Supplement: Supplementary file 1 [file Data_Sheet_1.PDF]

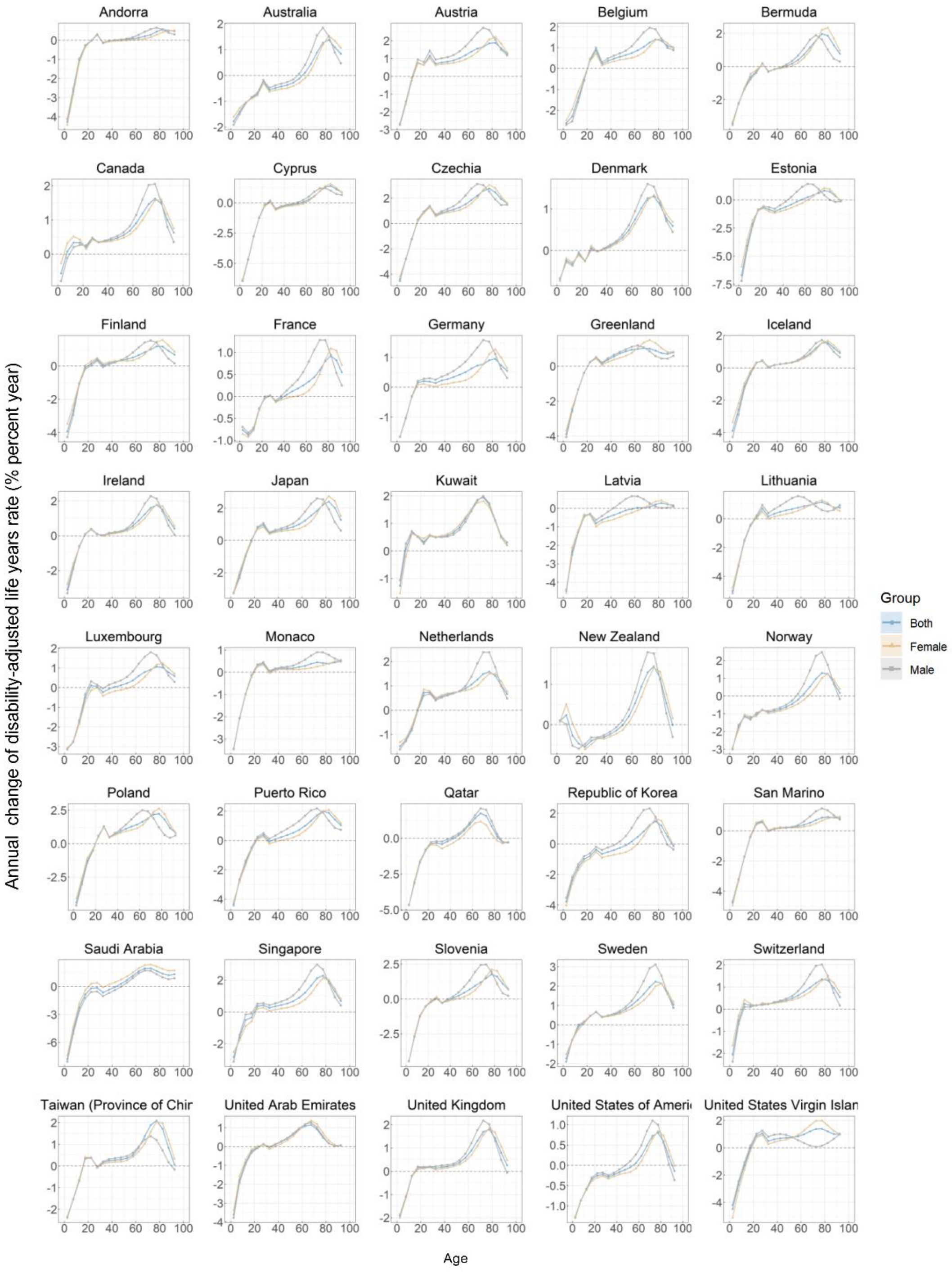

Figure S1 The local drifts of neonatal preterm birth rate in High SDI countries, 1992-2021. The dots and shaded areas indicate the values of local drift (annual percentage change in disability-adjusted life years rate) and its 95% CIs for neonatal preterm birth in 19 age groups (0-4 to 90-94 years). SDI=Socio-demographic Index.

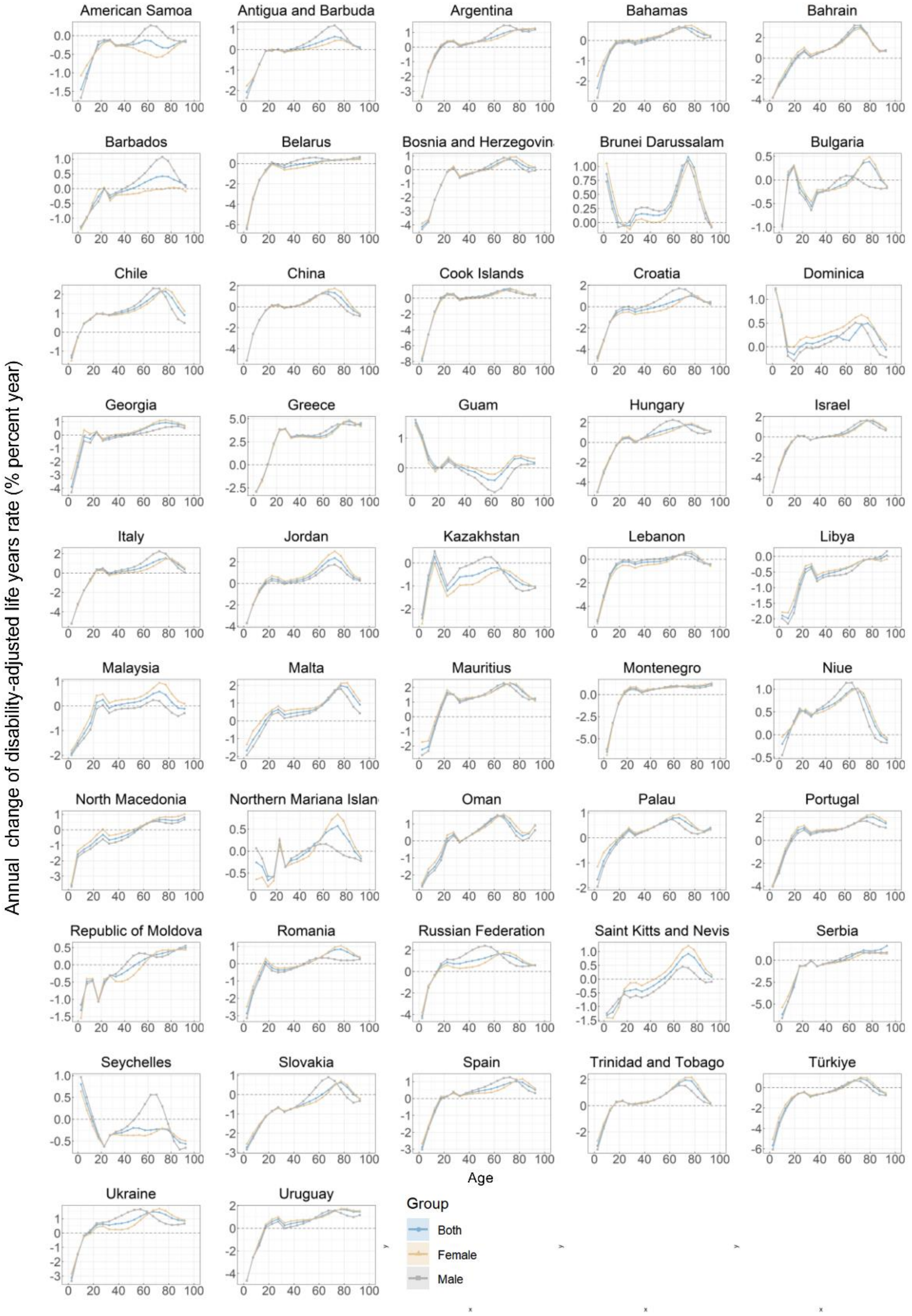

Figure S2 The local drifts of neonatal preterm birth rate in High-middle SDI countries, 1992-2021. The dots and shaded areas indicate the values of local drift (annual percentage change in disability-adjusted life years rate) and its 95% CIs for neonatal preterm birth in 19 age groups (0-4 to 90-94 years). SDI=Socio-demographic Index.

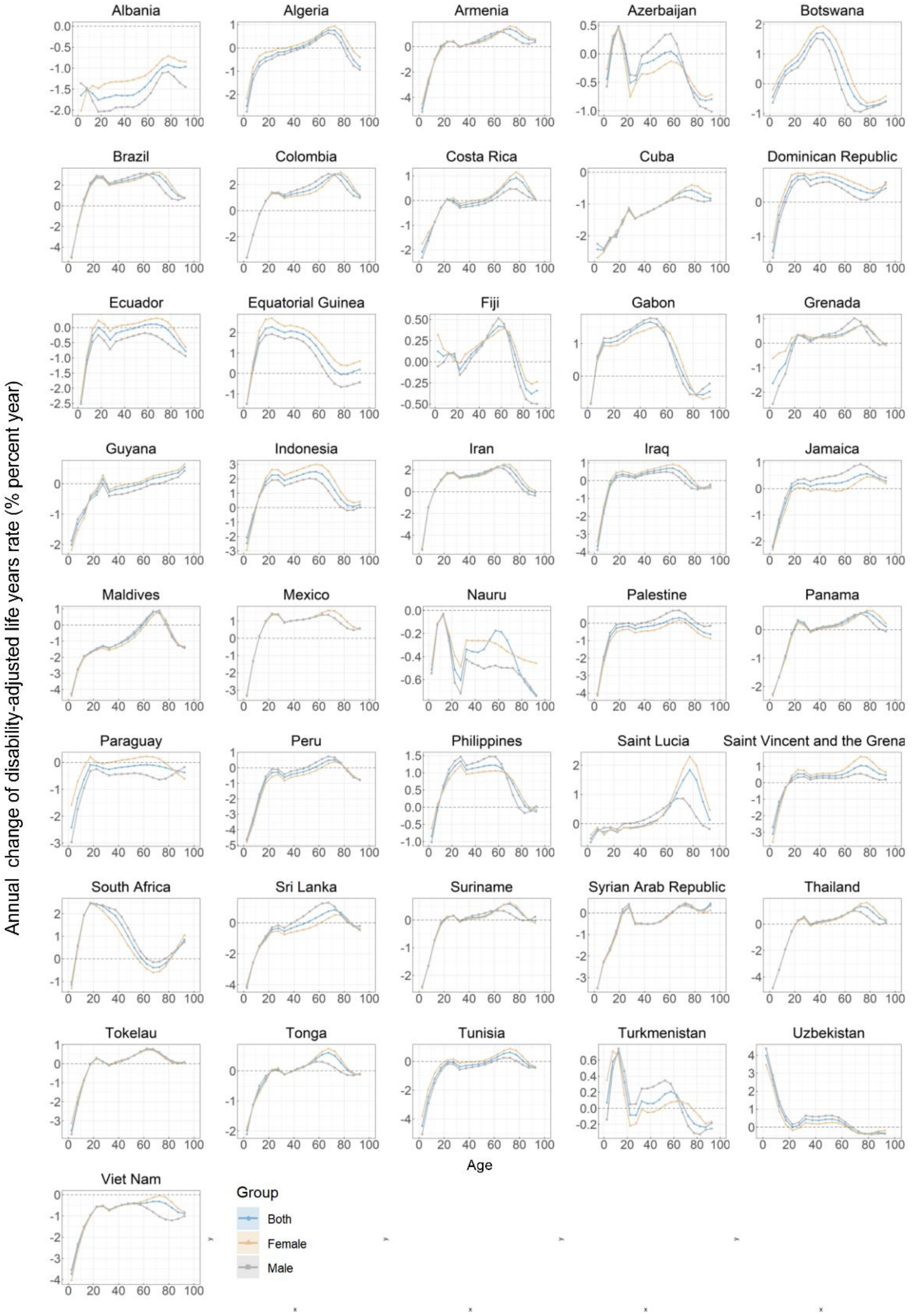

Figure S3 The local drifts of neonatal preterm birth disability-adjusted life years rate in Middle SDI countries, 1992-2021. The dots and shaded areas indicate the values of local drift (annual percentage change in disability-adjusted life years rate) and its 95% CIs for neonatal preterm birth in 19 age groups (0-4 to 90-94 years). SDI=Socio-demographic Index.

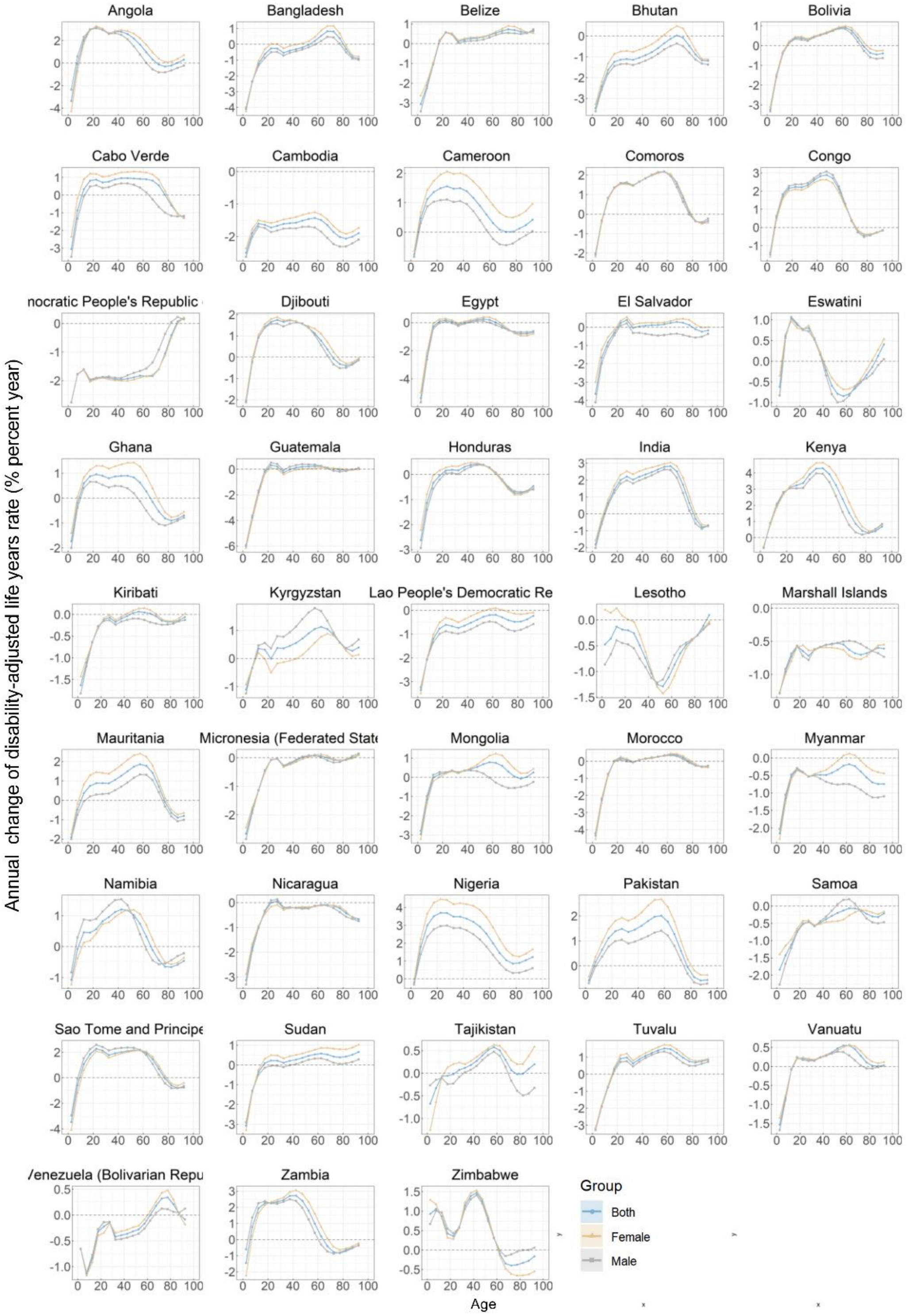

Figure S4 The local drifts of neonatal preterm birth disability-adjusted life years rate in Low-middle SDI countries, 1992-2021. The dots and shaded areas indicate the values of local drift (annual percentage change in disability-adjusted life years rate) and its 95% CIs for neonatal preterm birth in 19 age groups (0-4 to 90-94 years). SDI=Socio-demographic Index.

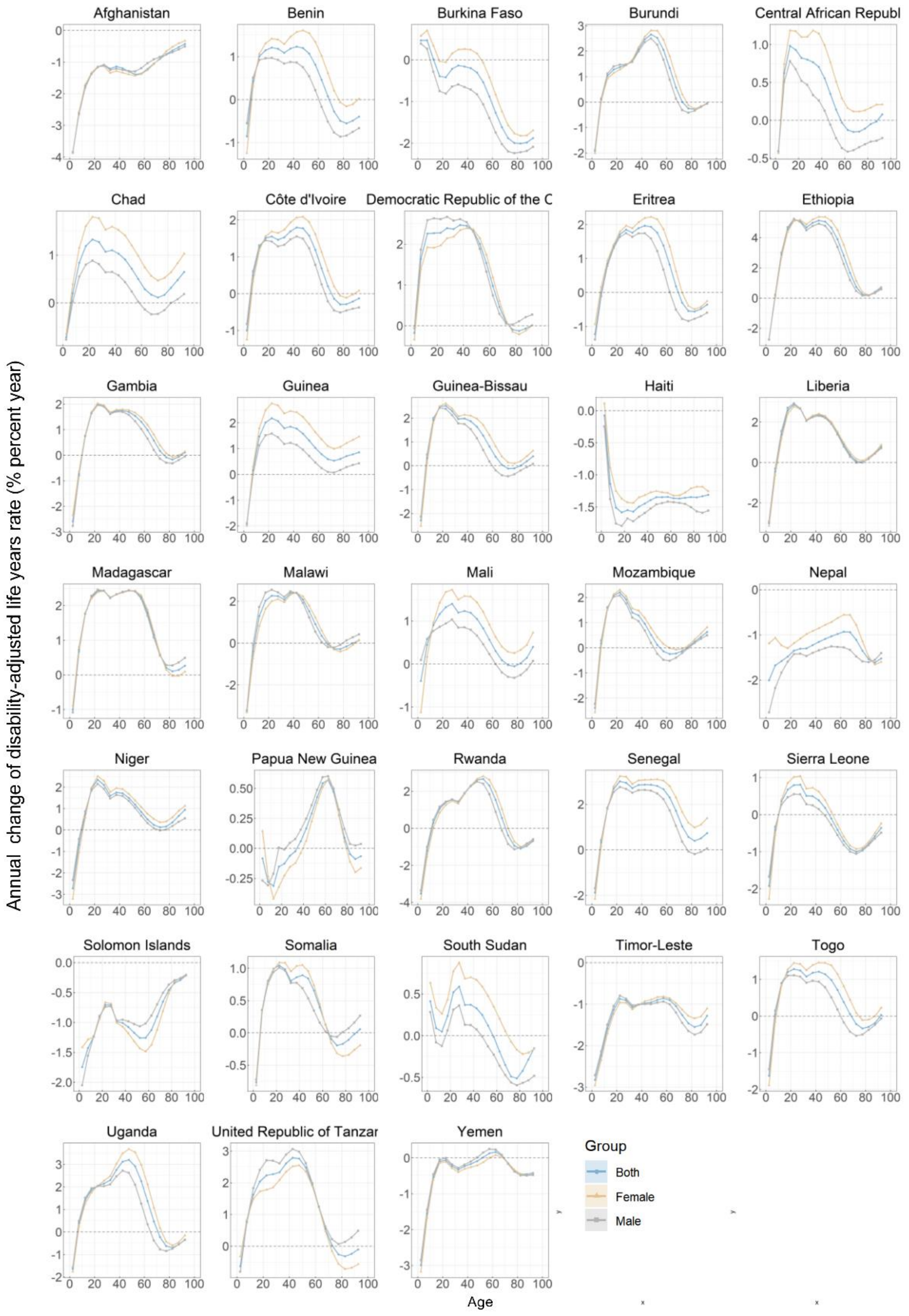

Figure S5 The local drifts of neonatal preterm birth disability-adjusted life years rate in Low SDI countries, 1992-2021. The dots and shaded areas indicate the values of local drift (annual percentage change in disability-adjusted life years rate) and its 95% CIs for neonatal preterm birth in 19 age groups (0-4 to 90-94 years). SDI=Socio-demographic Index.

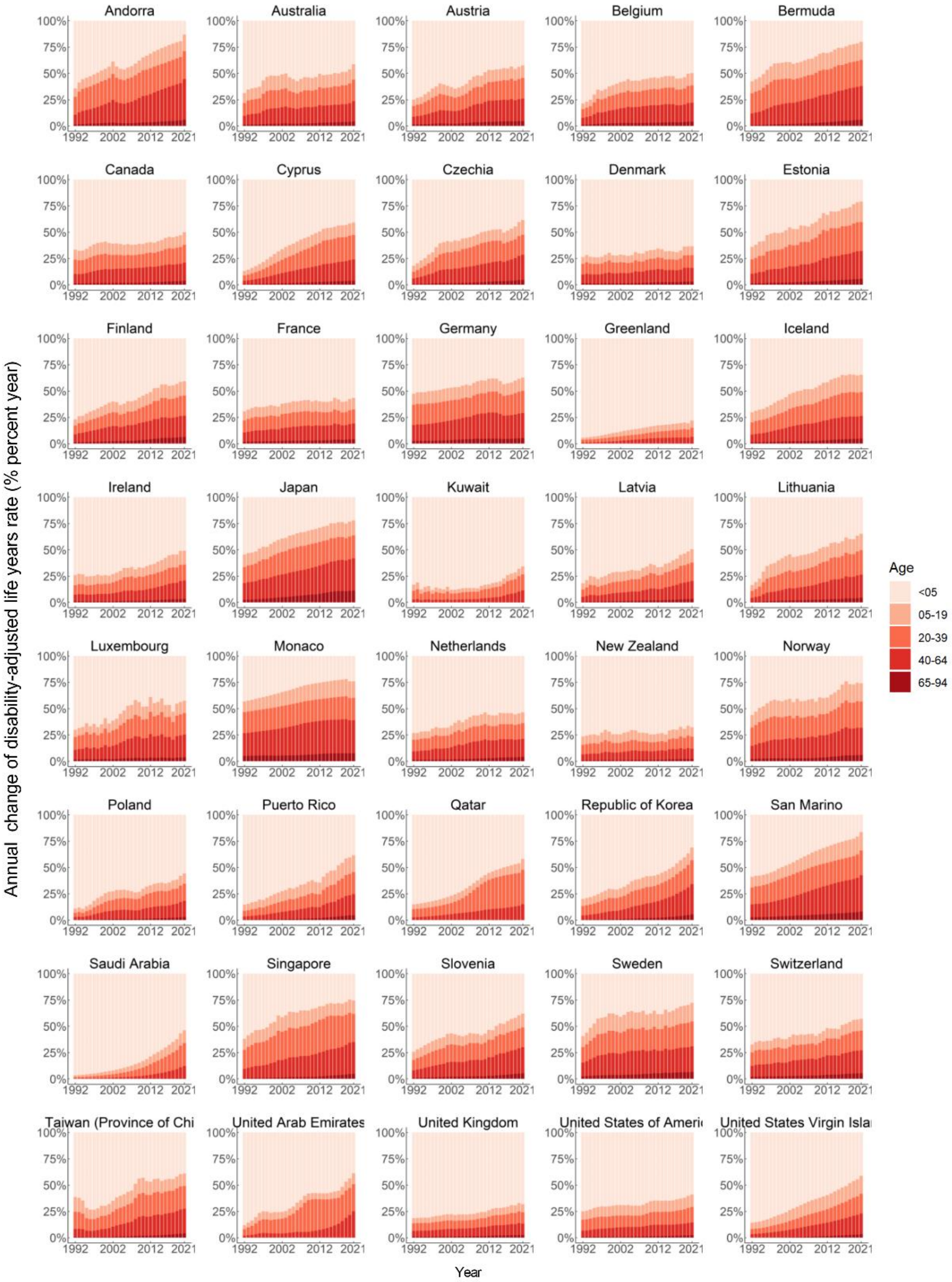

Figure S6 Age distribution of disability-adjusted life years from neonatal preterm birth in High SDI countries, 1992-2021. Age distribution of disability-adjusted life years is represented as temporal change in the relative proportion of disability-adjusted life years across age groups (<5, 5-19, 20-39, 40-64, 65-94 years) during 1992-2021. SDI=Socio-demographic Index.

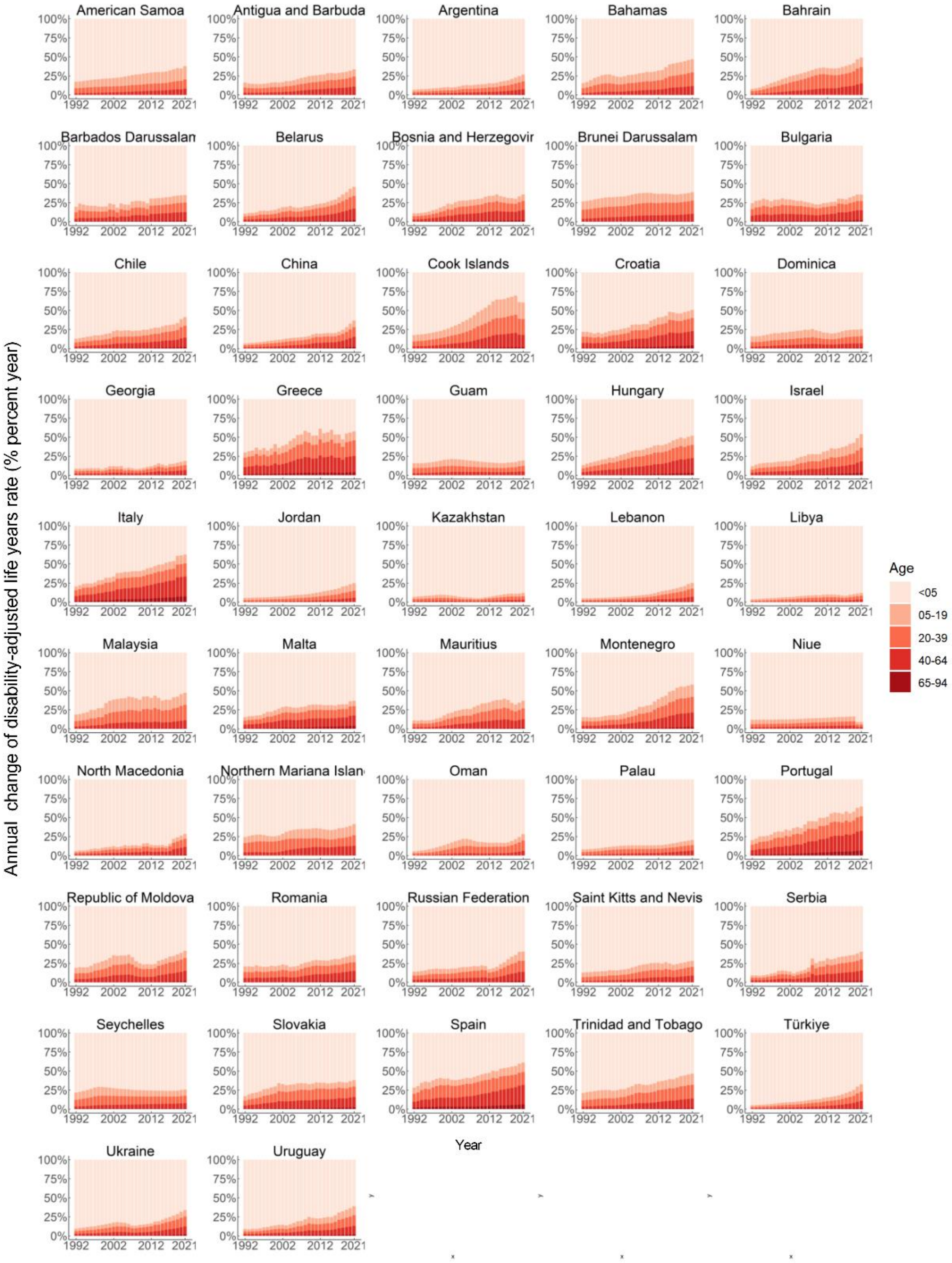

Figure S7 Age distribution of disability-adjusted life years from neonatal preterm birth in High-middle SDI countries, 1992-2021. Age distribution of disability-adjusted life years is represented as temporal change in the relative proportion of disability-adjusted life years across age groups (<5, 5-19, 20-39, 40-64, 65-94 years) during 1992-2021. SDI=Socio-demographic Index.

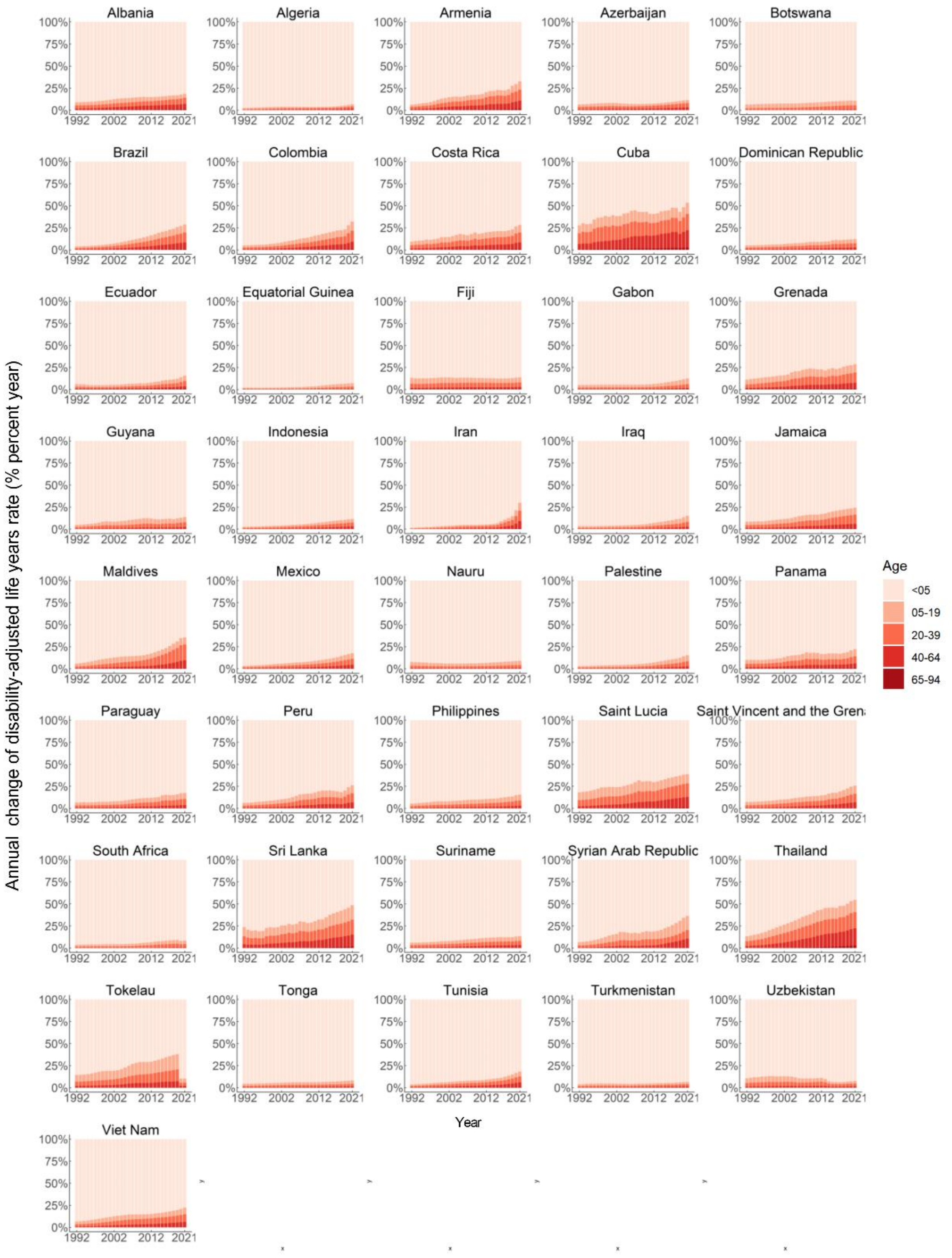

Figure S8 Age distribution of disability-adjusted life years from neonatal preterm birth in Middle SDI countries, 1992-2021. Age distribution of disability-adjusted life years is represented as temporal change in the relative proportion of disability-adjusted life years across age groups (<5, 5-19, 20-39, 40-64, 65-94 years) during 1992-2021. SDI=Socio-demographic Index.

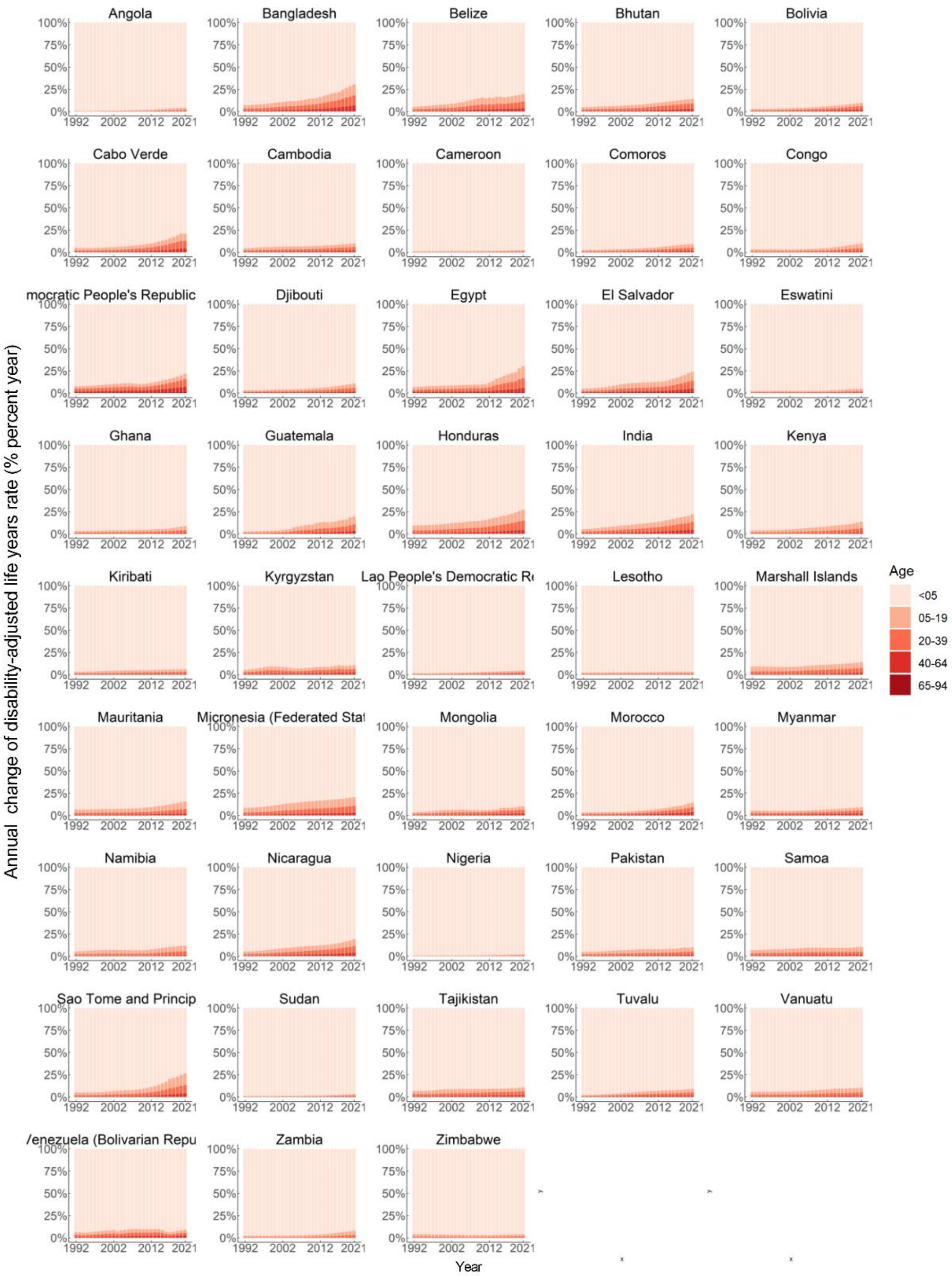

Figure S9 Age distribution of disability-adjusted life years from neonatal preterm birth in Low-middle SDI countries, 1992-2021. Age distribution of disability-adjusted life years is represented as temporal change in the relative proportion of disability-adjusted life years across age groups (<5, 5-19, 20-39, 40-64, 65-94 years) during 1992-2021. SDI=Socio-demographic Index.

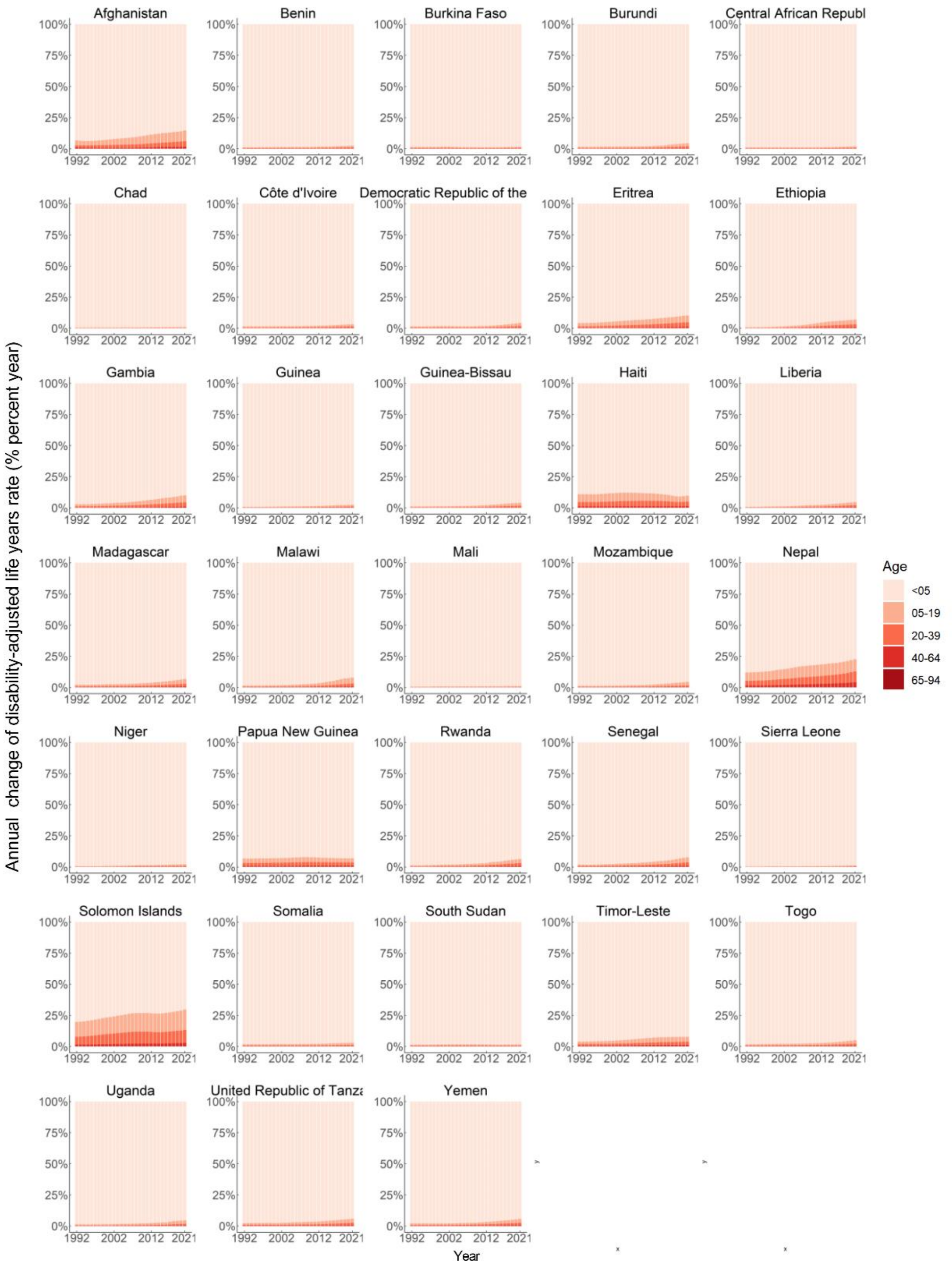

Figure S10 Age distribution of disability-adjusted life years from neonatal preterm birth in Low SDI countries, 1992-2021. Age distribution of disability-adjusted life years is represented as temporal change in the relative proportion of disability-adjusted life years across age groups (<5, 5-19, 20-39, 40-64, 65-94 years) during 1992-2021. SDI=Socio-demographic Index.

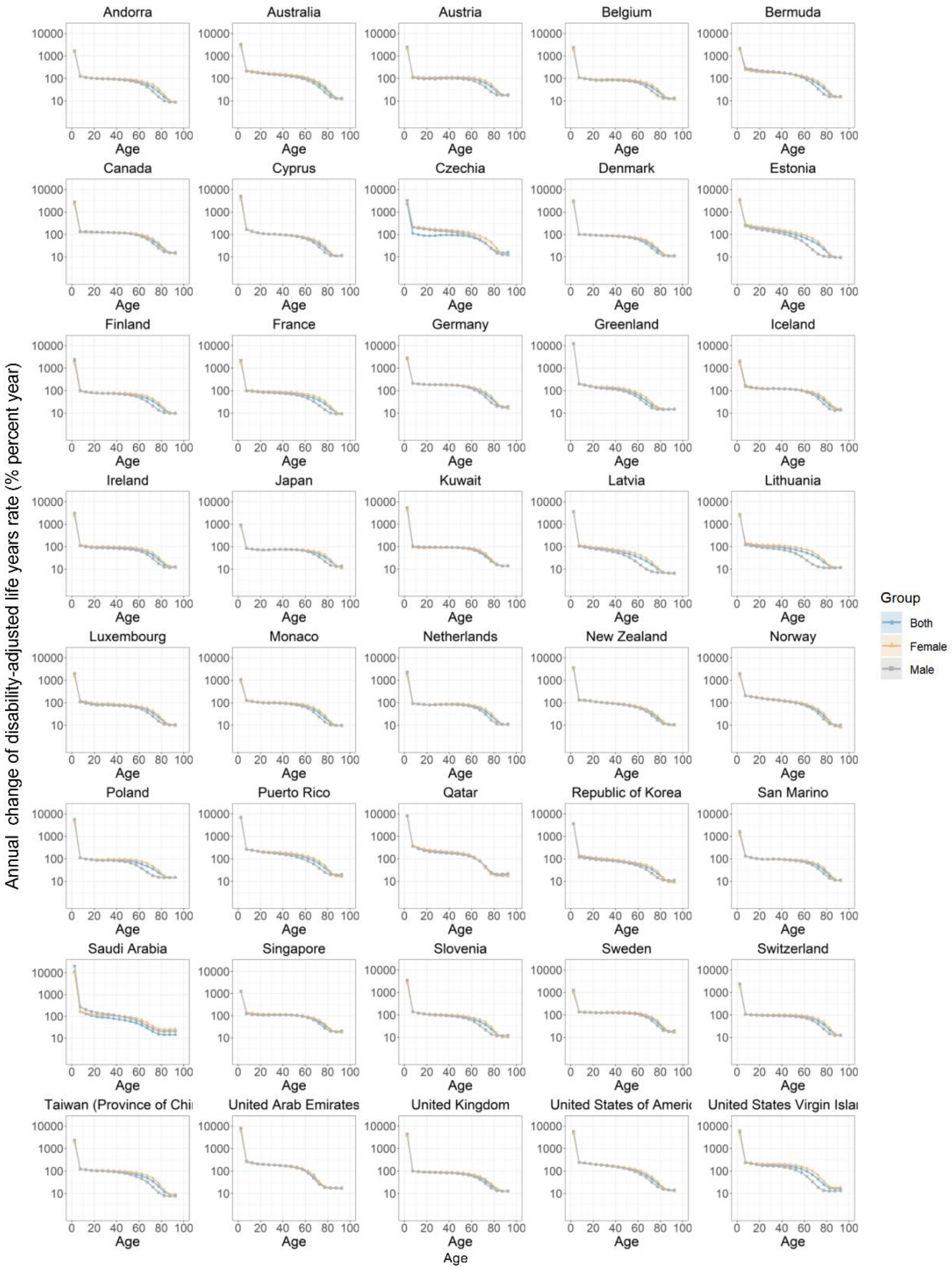

Figure S11 Age effects on neonatal preterm birth disability-adjusted life years rate in High SDI countries. Age effects indicate age-associated natural history and are shown by the fitted longitudinal age curves of disability-adjusted life years rate (per 100000 person-years) adjusted for period deviations, with the dots and shaded areas denoting disability-adjusted life years rates with 95% CIs. SDI=Socio-demographic Index.

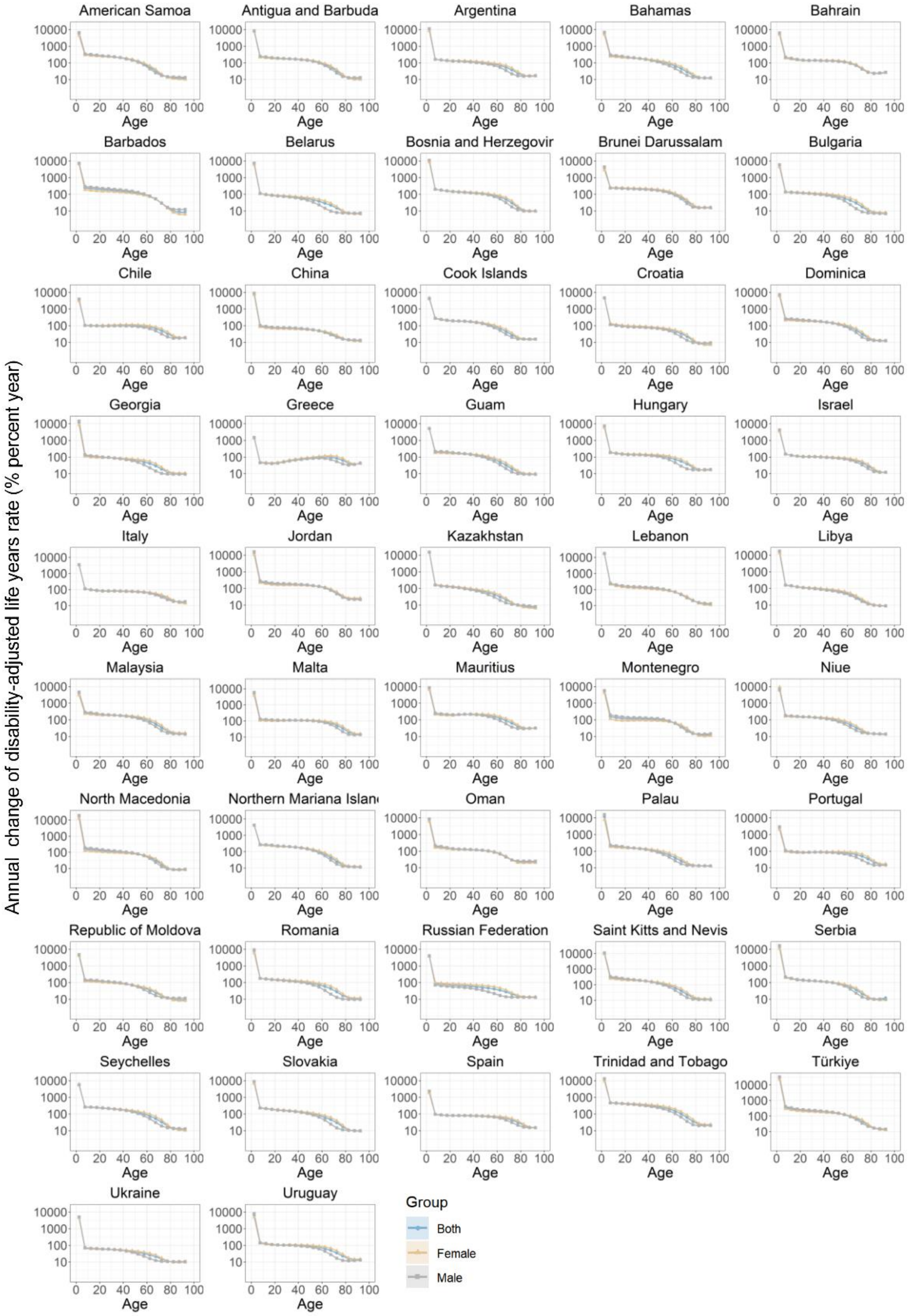

Figure S12 Age effects on neonatal preterm birth disability-adjusted life years rate in High-middle SDI countries. Age effects indicate age-associated natural history and are shown by the fitted longitudinal age curves of disability-adjusted life years rate (per 100000 person-years) adjusted for period deviations, with the dots and shaded areas denoting disability-adjusted life years rates with 95% CIs. SDI=Socio-demographic Index.

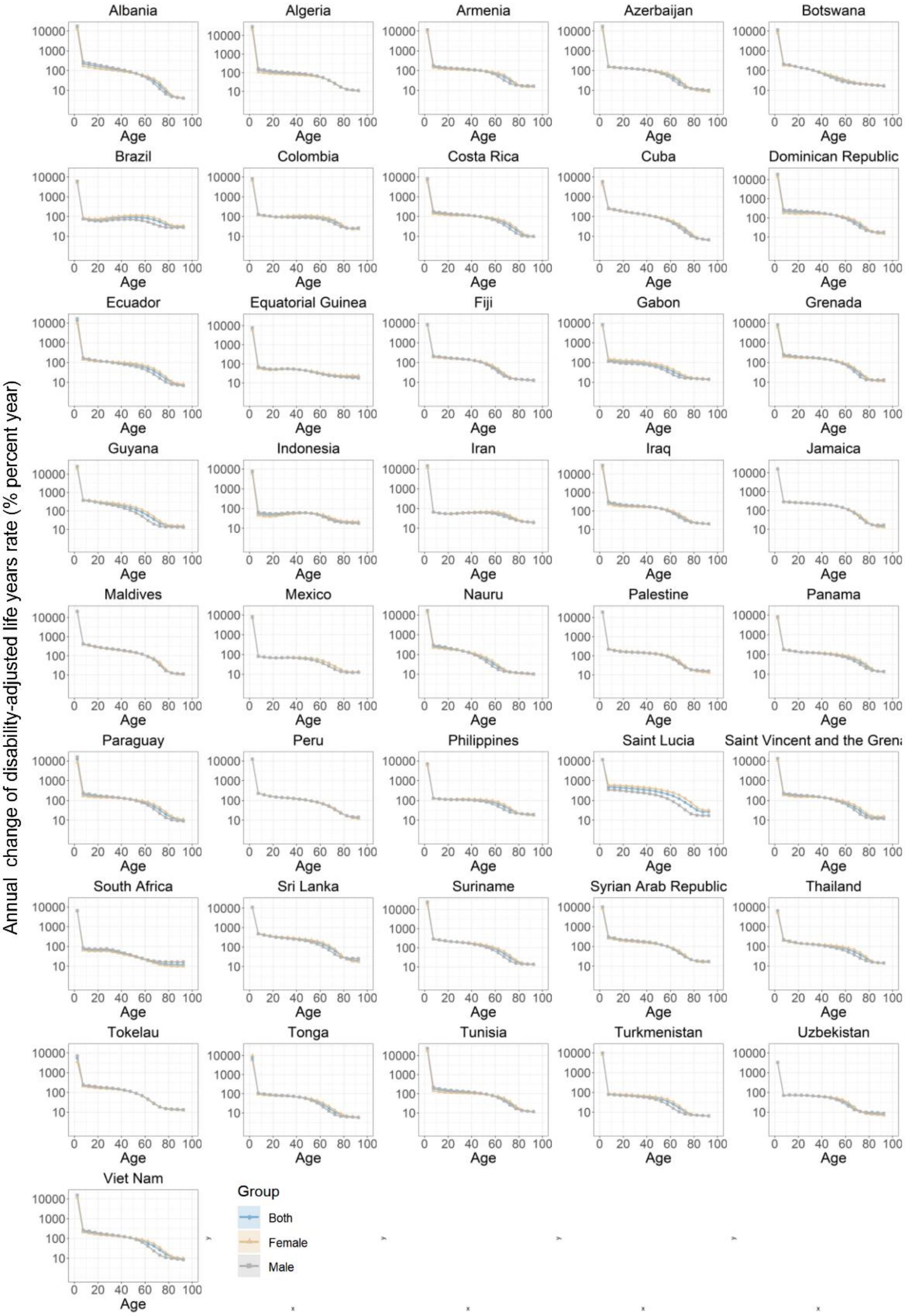

Figure S13 Age effects on neonatal preterm birth disability-adjusted life years rate in Middle SDI countries. Age effects indicate age-associated natural history and are shown by the fitted longitudinal age curves of disability-adjusted life years rate (per 100000 person-years) adjusted for period deviations, with the dots and shaded areas denoting disability-adjusted life years rates with 95% CIs. SDI=Socio-demographic Index.

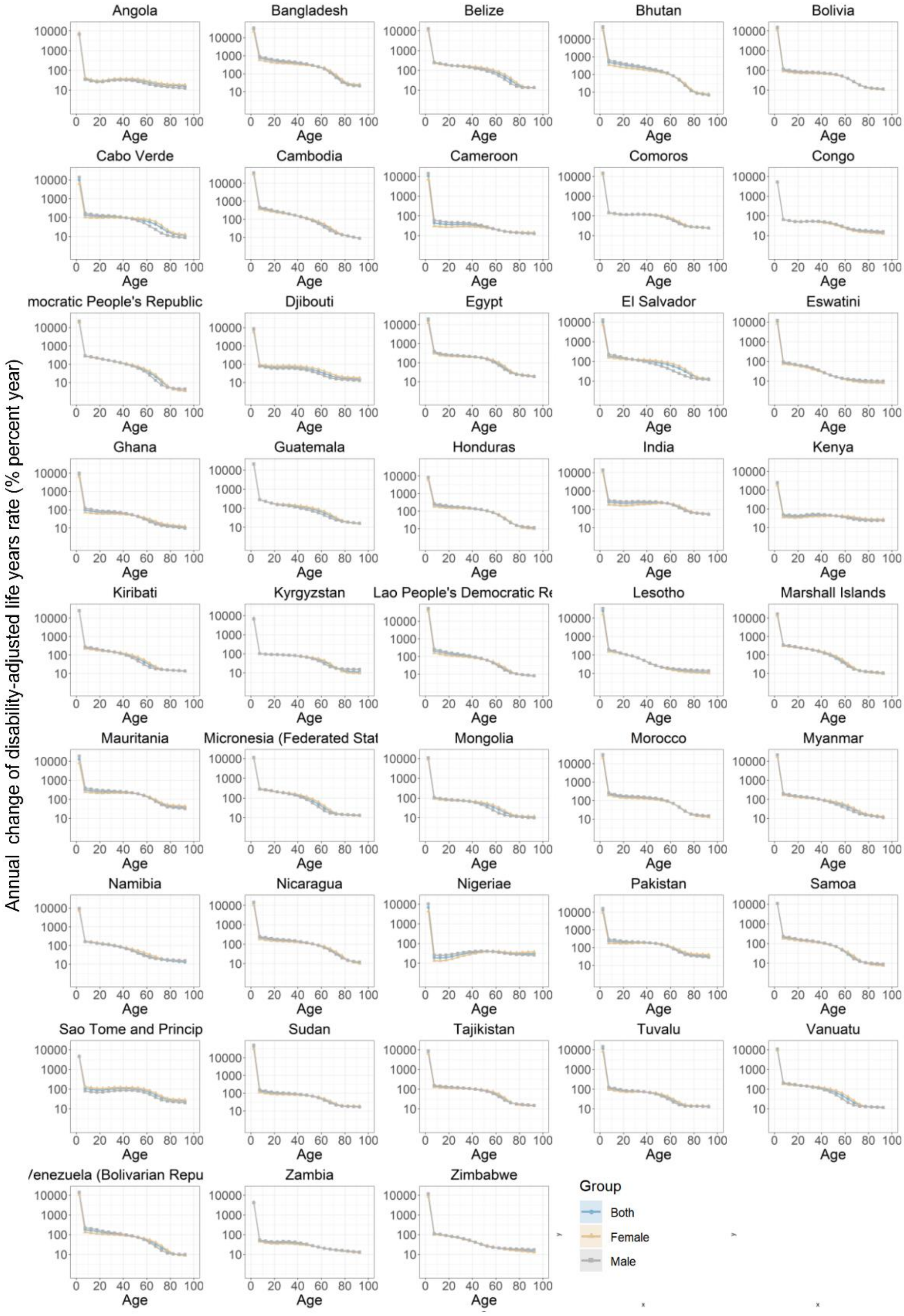

Figure S14 Age effects on neonatal preterm birth disability-adjusted life years rate in Low-middle SDI countries. Age effects indicate age-associated natural history and are shown by the fitted longitudinal age curves of disability-adjusted life years rate (per 100000 person-years) adjusted for period deviations, with the dots and shaded areas denoting disability-adjusted life years rates with 95% CIs. SDI=Socio-demographic Index.

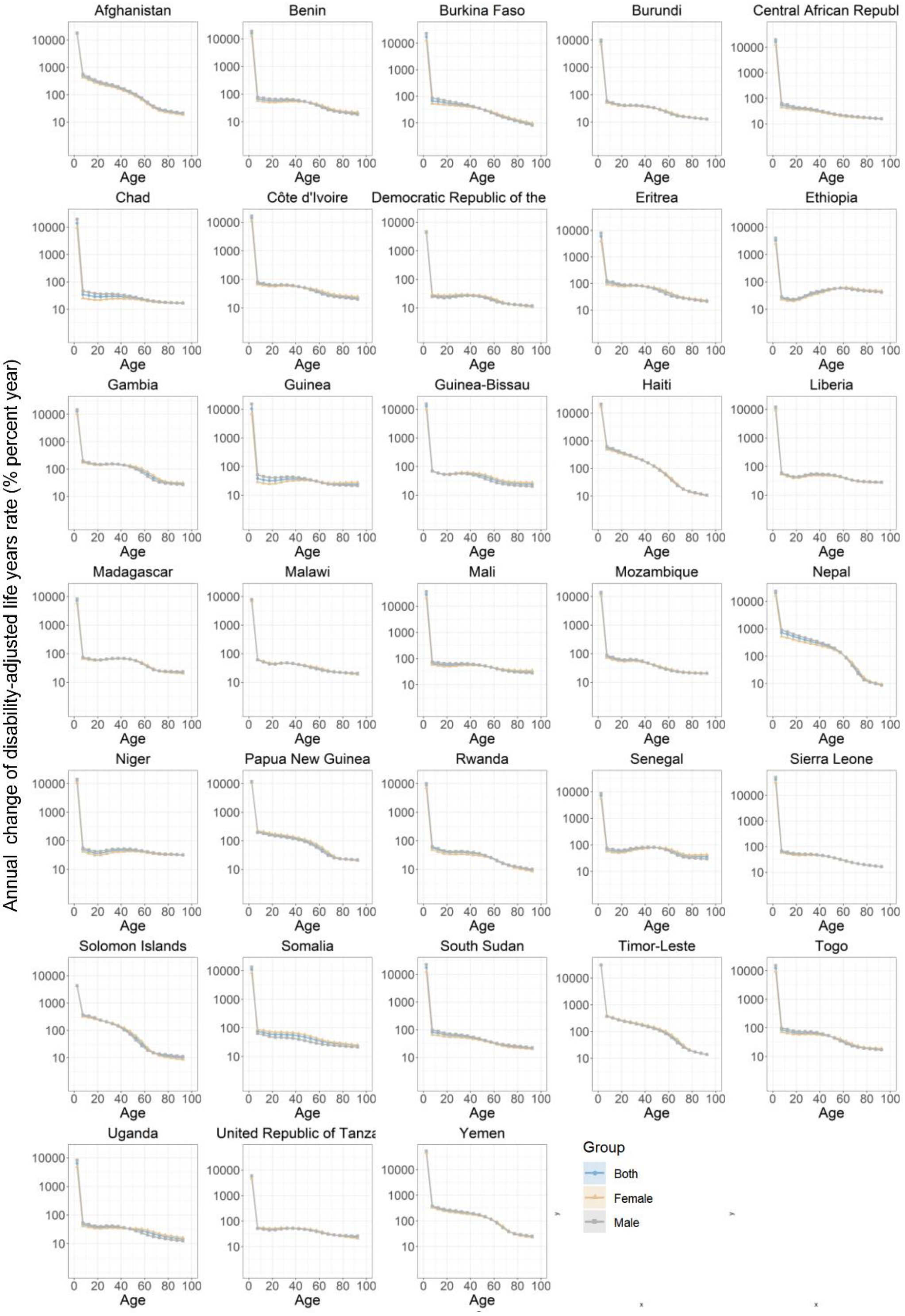

Figure S15 Age effects on neonatal preterm birth disability-adjusted life years rate in Low SDI countries. Age effects indicate age-associated natural history and are shown by the fitted longitudinal age curves of disability-adjusted life years rate (per 100000 person-years) adjusted for period deviations, with the dots and shaded areas denoting disability-adjusted life years rates with 95% CIs. SDI=Socio-demographic Index.

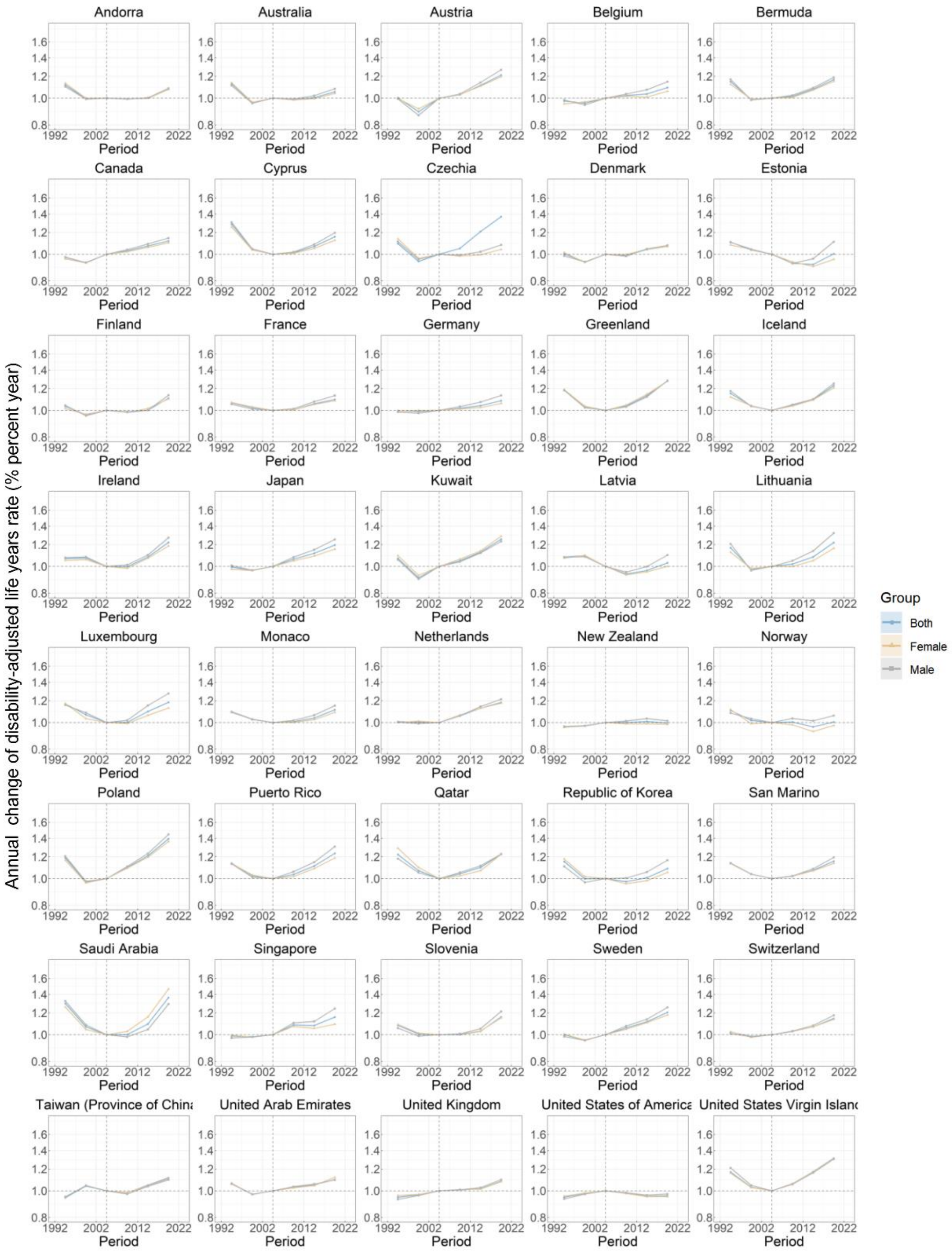

Figure S16 Period effects on neonatal preterm birth disability-adjusted life years rate in High SDI countries. Period effects are shown by the relative risk of disability-adjusted life years rate (disability-adjusted life years rate ratio) for each period from 1992-1996 to 2017-2021, with the dots and shaded areas representing rate ratios and 95% CIs for a given period relative to the referent period (2002-2006). SDI=Socio-demographic Index.

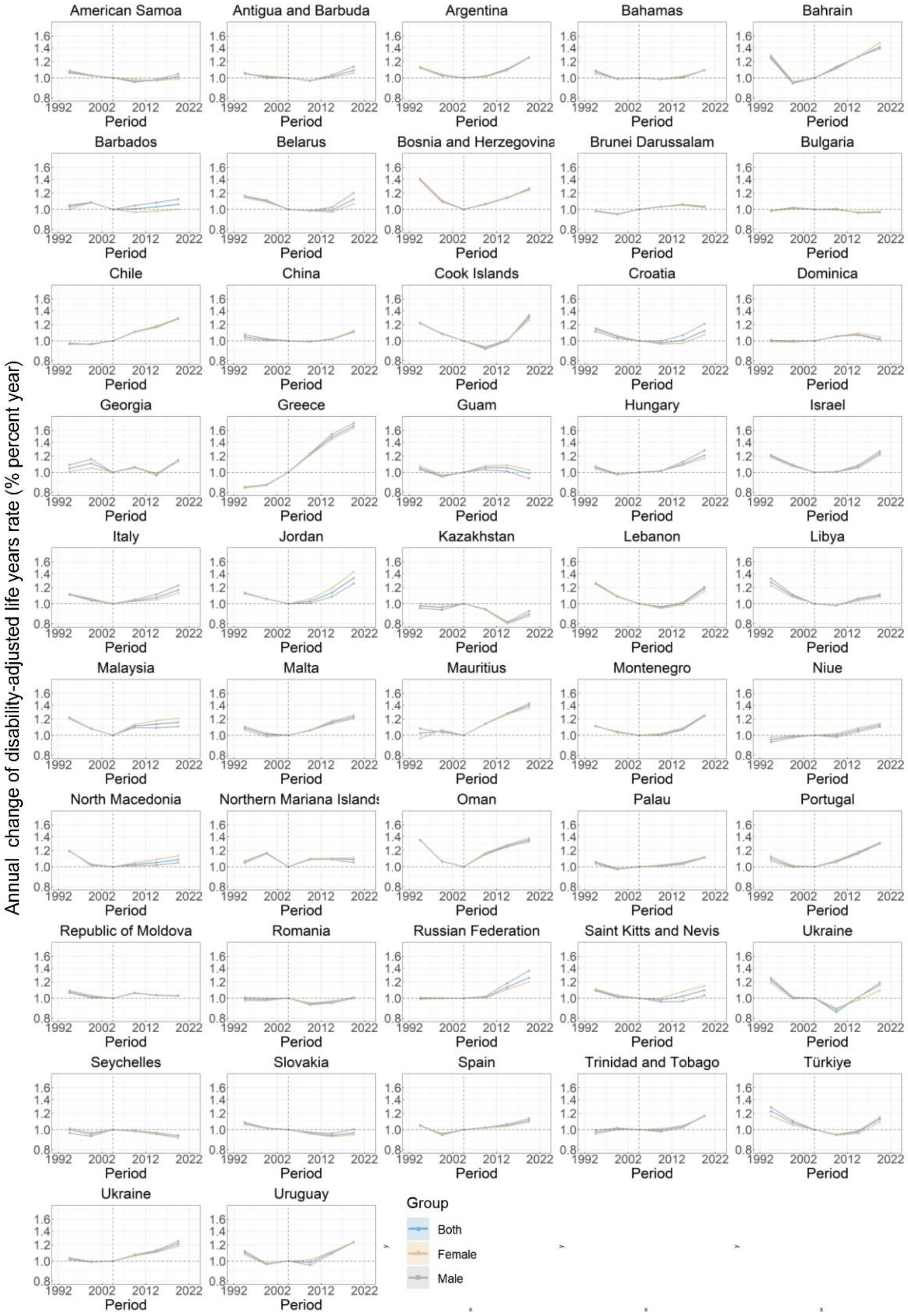

Figure S17 Period effects on neonatal preterm birth disability-adjusted life years rate in High-middle SDI countries. Period effects are shown by the relative risk of disability-adjusted life years rate (disability-adjusted life years rate ratio) for each period from 1992-1996 to 2017-2021, with the dots and shaded areas representing rate ratios and 95% CIs for a given period relative to the referent period (2002-2006). SDI=Socio-demographic Index.

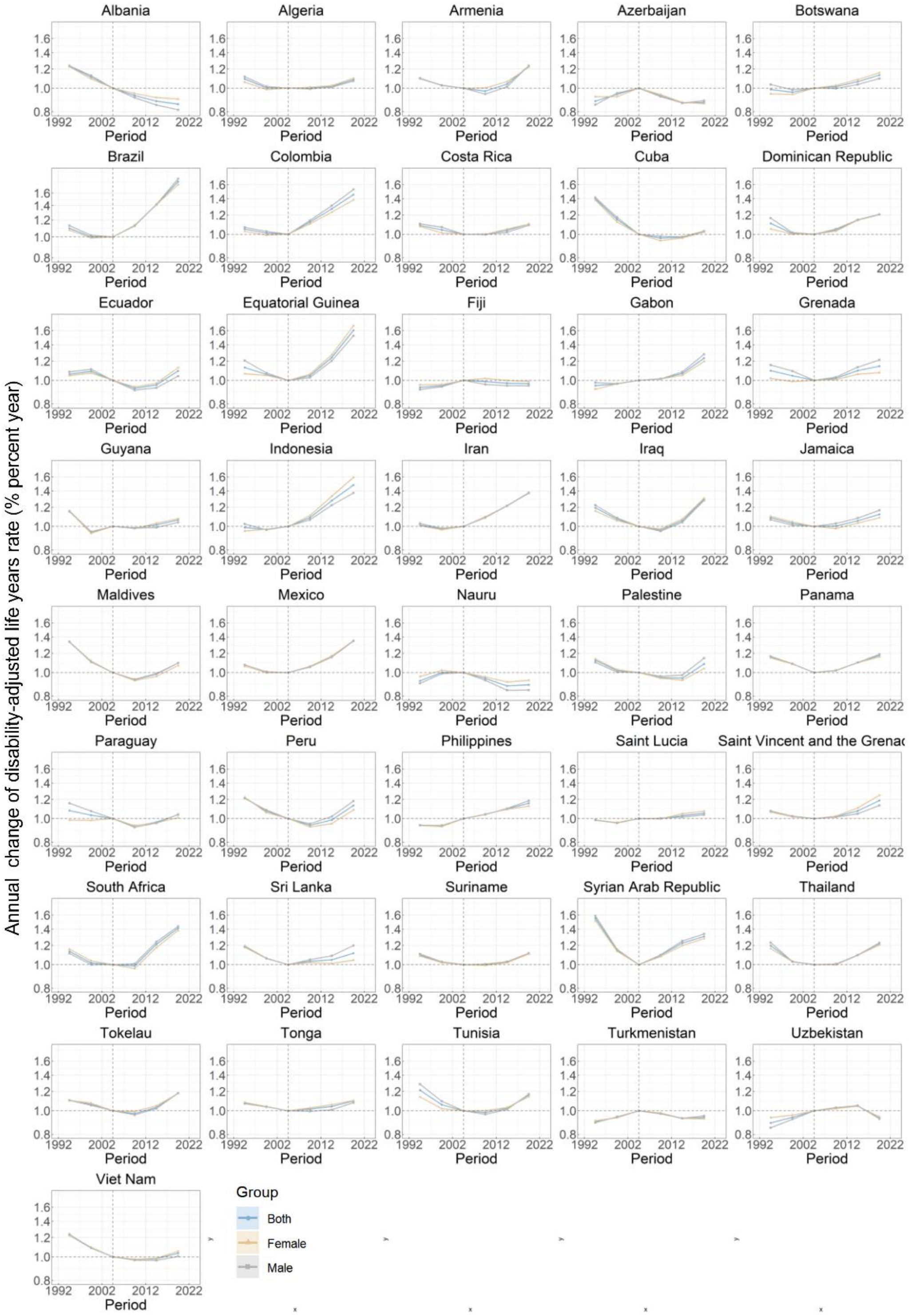

Figure S18 Period effects on neonatal preterm birth disability-adjusted life years rate in Middle SDI countries. Period effects are shown by the relative risk of disability-adjusted life years rate (disability-adjusted life years rate ratio) for each period from 1992-1996 to 2017-2021, with the dots and shaded areas representing rate ratios and 95% CIs for a given period relative to the referent period (2002-2006). SDI=Socio-demographic Index.

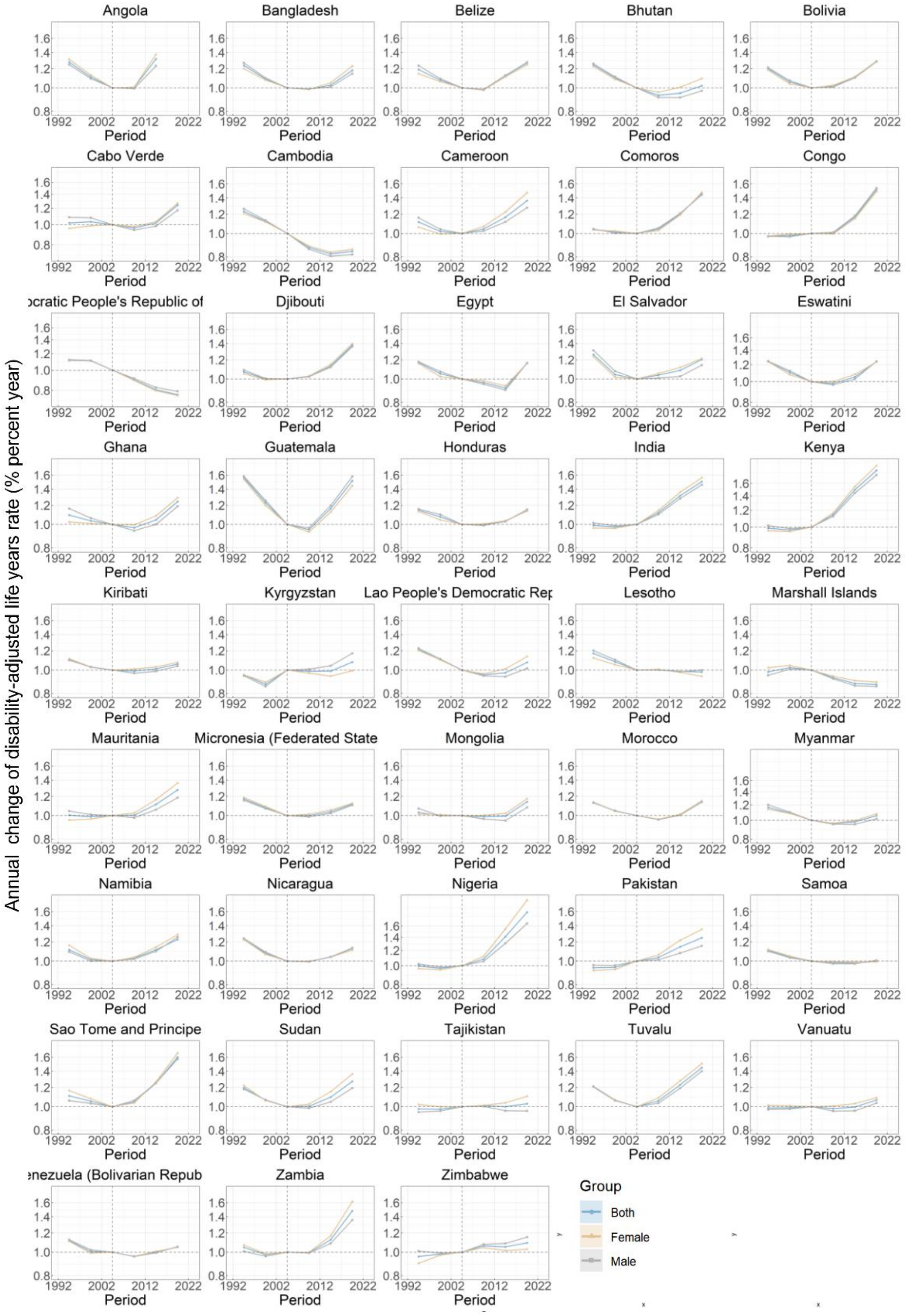

Figure S19 Period effects on neonatal preterm birth disability-adjusted life years rate in Low-middle SDI countries. Period effects are shown by the relative risk of disability-adjusted life years rate (disability-adjusted life years rate ratio) for each period from 1992-1996 to 2017-2021, with the dots and shaded areas representing rate ratios and 95% CIs for a given period relative to the referent period (2002-2006). SDI=Socio-demographic Index.

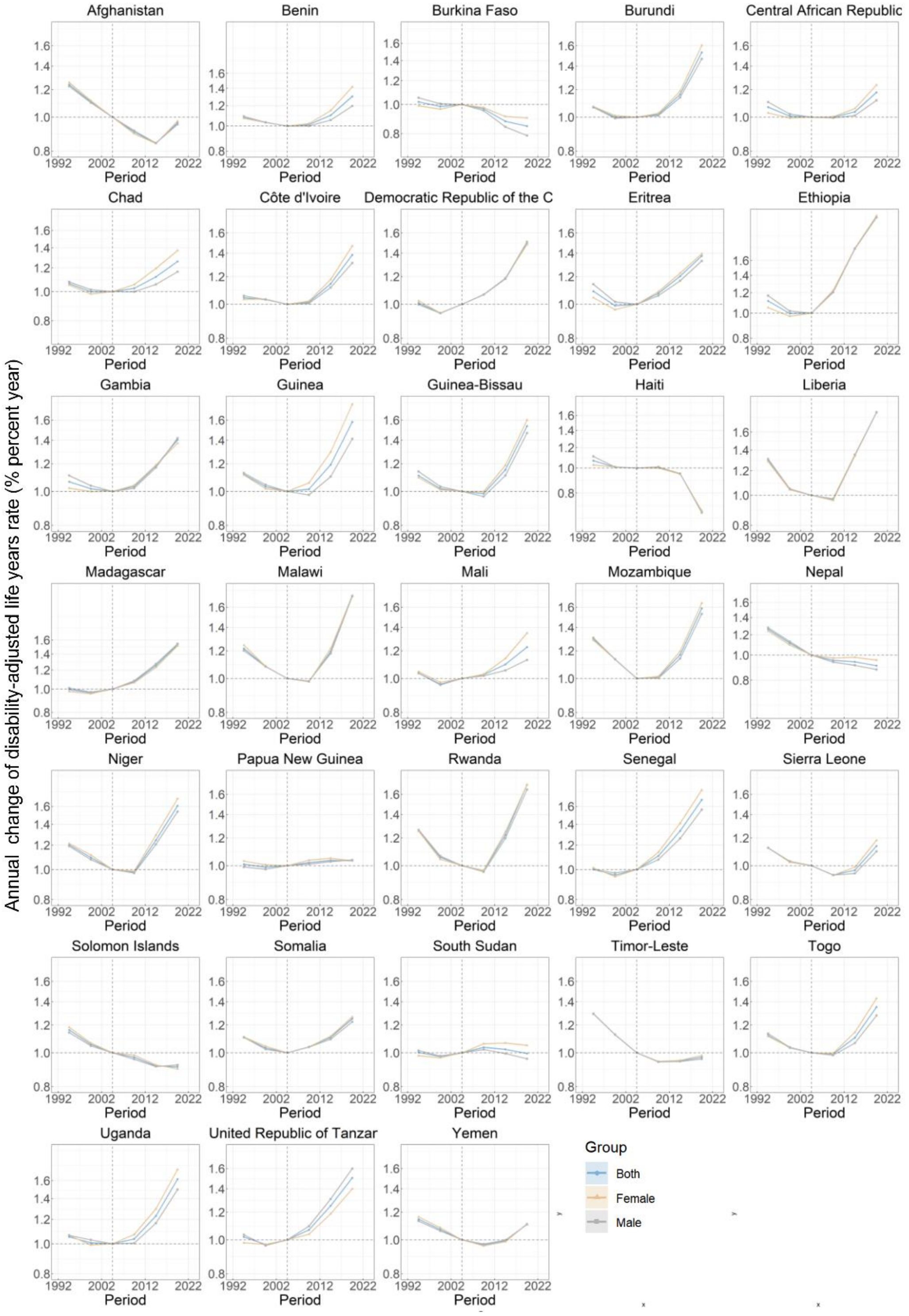

Figure S20 Period effects on neonatal preterm birth disability-adjusted life years rate in Low SDI countries. Period effects are shown by the relative risk of disability-adjusted life years rate (disability-adjusted life years rate ratio) for each period from 1992-1996 to 2017-2021, with the dots and shaded areas representing rate ratios and 95% CIs for a given period relative to the referent period (2002-2006). SDI=Socio-demographic Index.

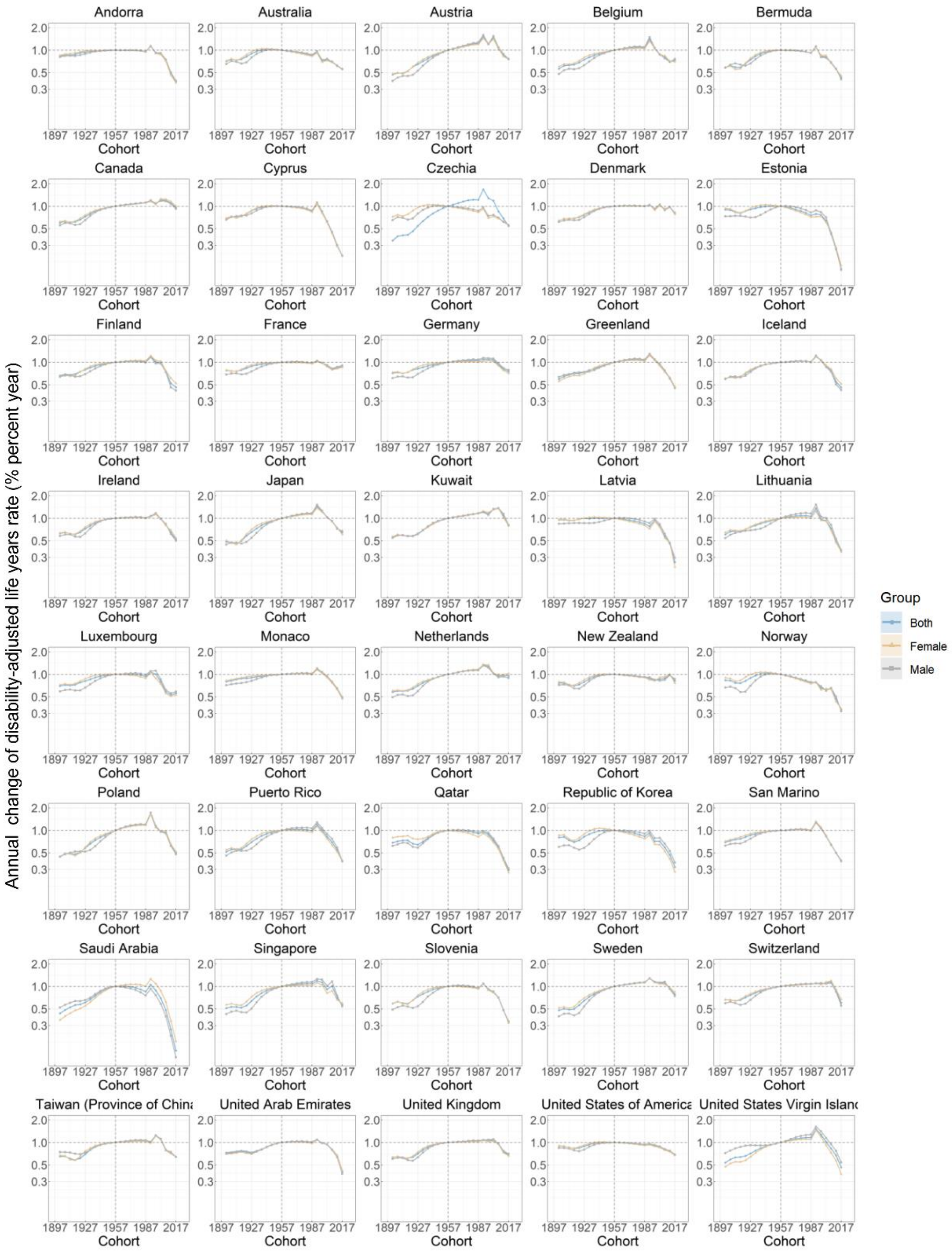

Figure S21 Cohort effects on neonatal preterm birth disability-adjusted life years rate in High SDI countries. Cohort effects are shown by the relative risk of disability-adjusted life years rate (disability-adjusted life years rate ratio) for each birth cohort from 1902 to 2017, with the dots and shaded areas represent rate ratios and 95% CIs for a given cohort relative to the referent 1957 cohort. SDI=Socio-demographic Index.

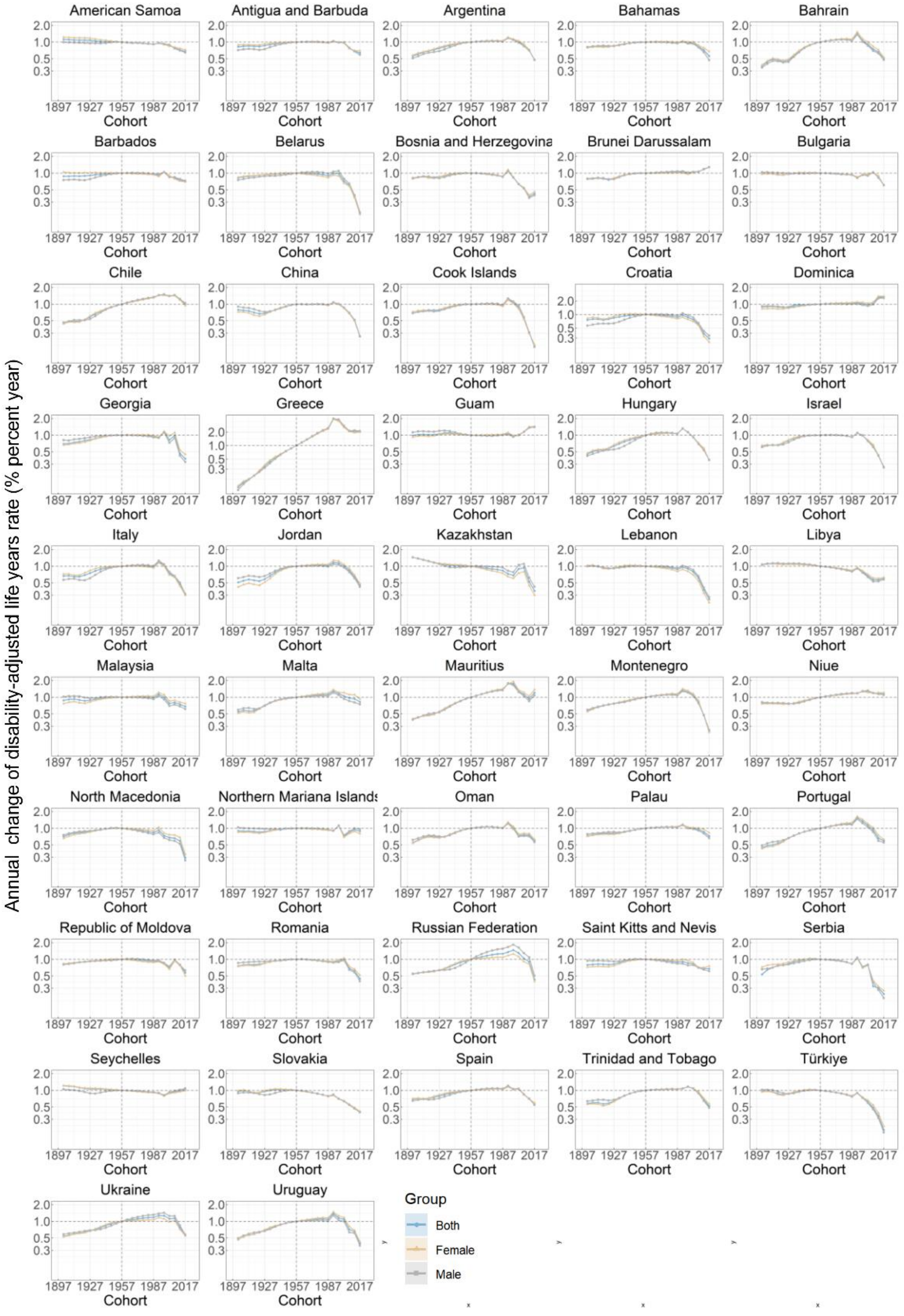

Figure S22 Cohort effects on neonatal preterm birth disability-adjusted life years rate in High-middle SDI countries. Cohort effects are shown by the relative risk of disability-adjusted life years rate (disability-adjusted life years rate ratio) for each birth cohort from 1902 to 2017, with the dots and shaded areas represent rate ratios and 95% CIs for a given cohort relative to the referent 1957 cohort. SDI=Socio-demographic Index.

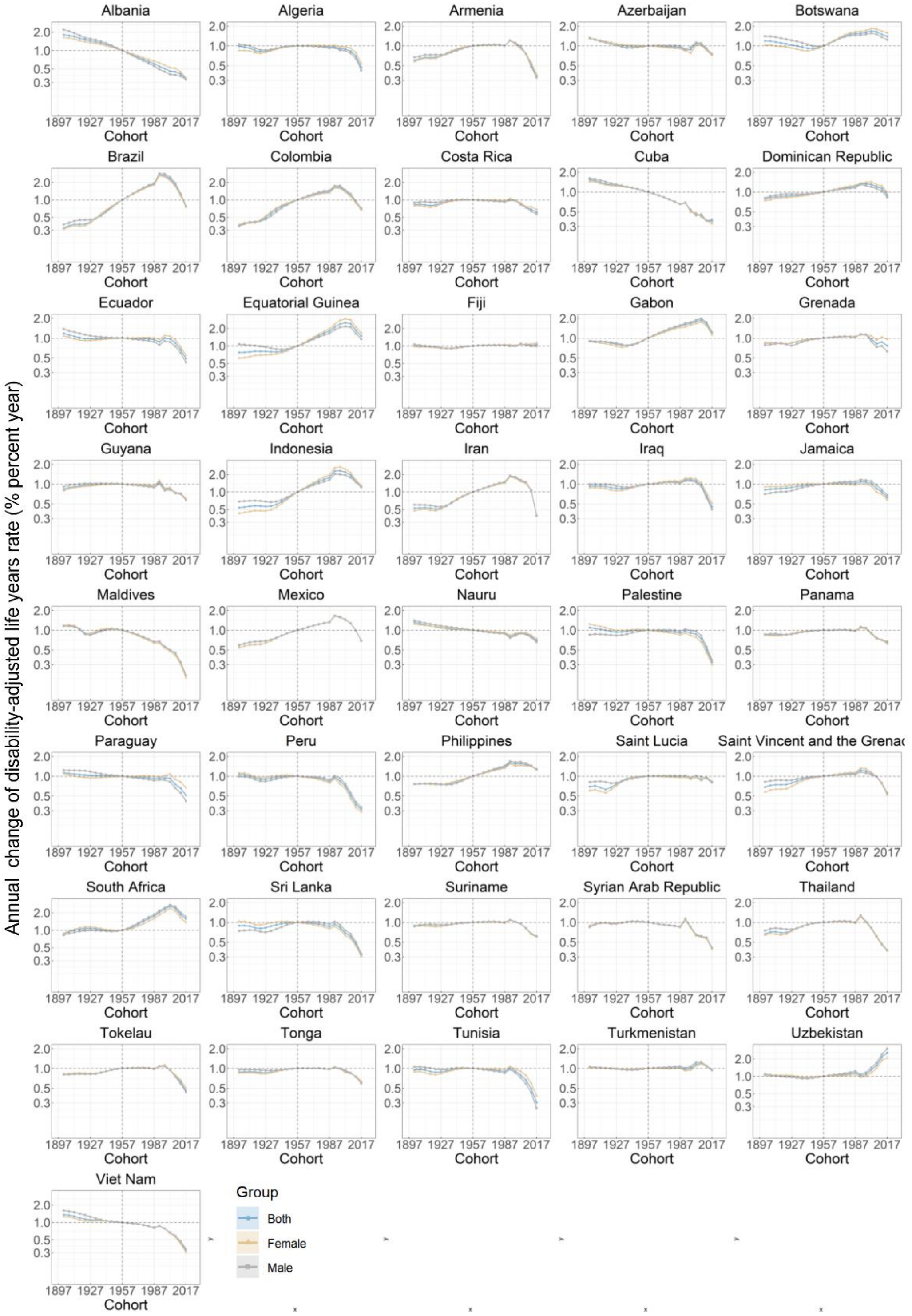

Figure S23 Cohort effects on neonatal preterm birth disability-adjusted life years rate in Middle SDI countries. Cohort effects are shown by the relative risk of disability-adjusted life years rate (disability-adjusted life years rate ratio) for each birth cohort from 1902 to 2017, with the dots and shaded areas represent rate ratios and 95% CIs for a given cohort relative to the referent 1957 cohort. SDI=Socio-demographic Index.

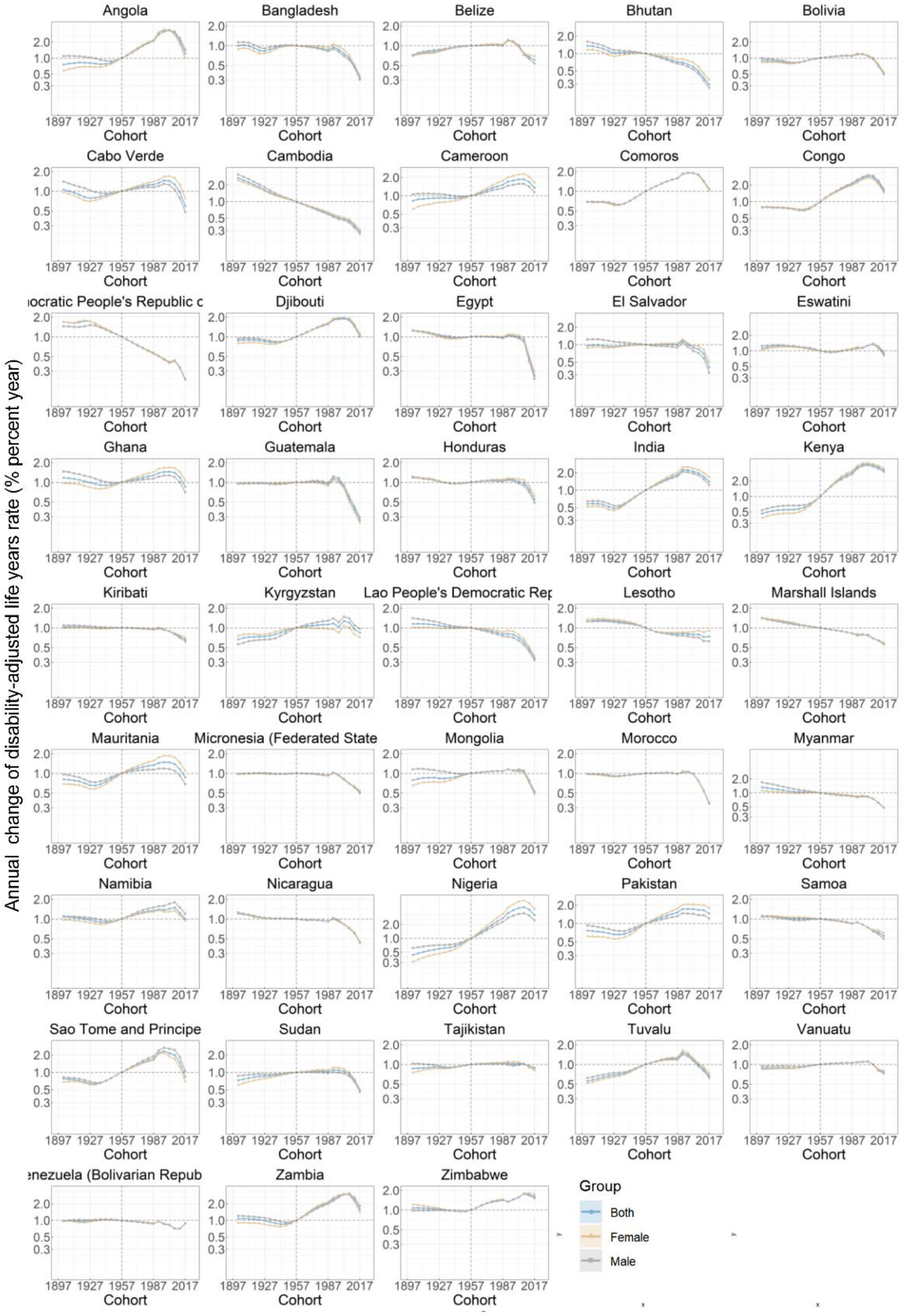

Figure S24 Cohort effects on neonatal preterm birth disability-adjusted life years rate in Low-middle SDI countries. Cohort effects are shown by the relative risk of disability-adjusted life years rate (disability-adjusted life years rate ratio) for each birth cohort from 1902 to 2017, with the dots and shaded areas represent rate ratios and 95% CIs for a given cohort relative to the referent 1957 cohort. SDI=Socio-demographic Index.

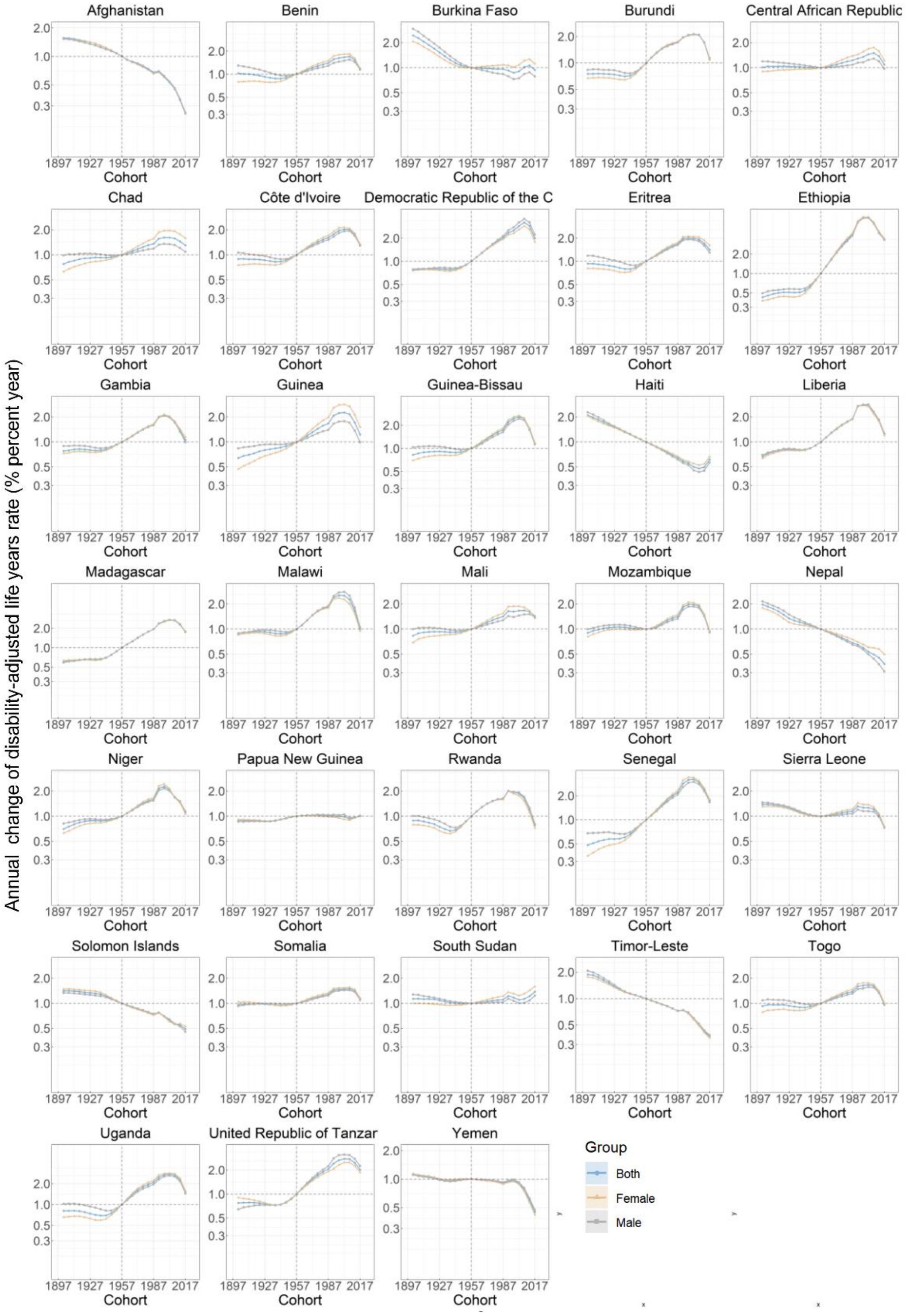

Figure S25 Cohort effects on neonatal preterm birth disability-adjusted life years rate in Low SDI countries. Cohort effects are shown by the relative risk of disability-adjusted life years rate (disability-adjusted life years rate ratio) for each birth cohort from 1902 to 2017, with the dots and shaded areas represent rate ratios and 95% CIs for a given cohort relative to the referent 1957 cohort. SDI=Socio-demographic Index.

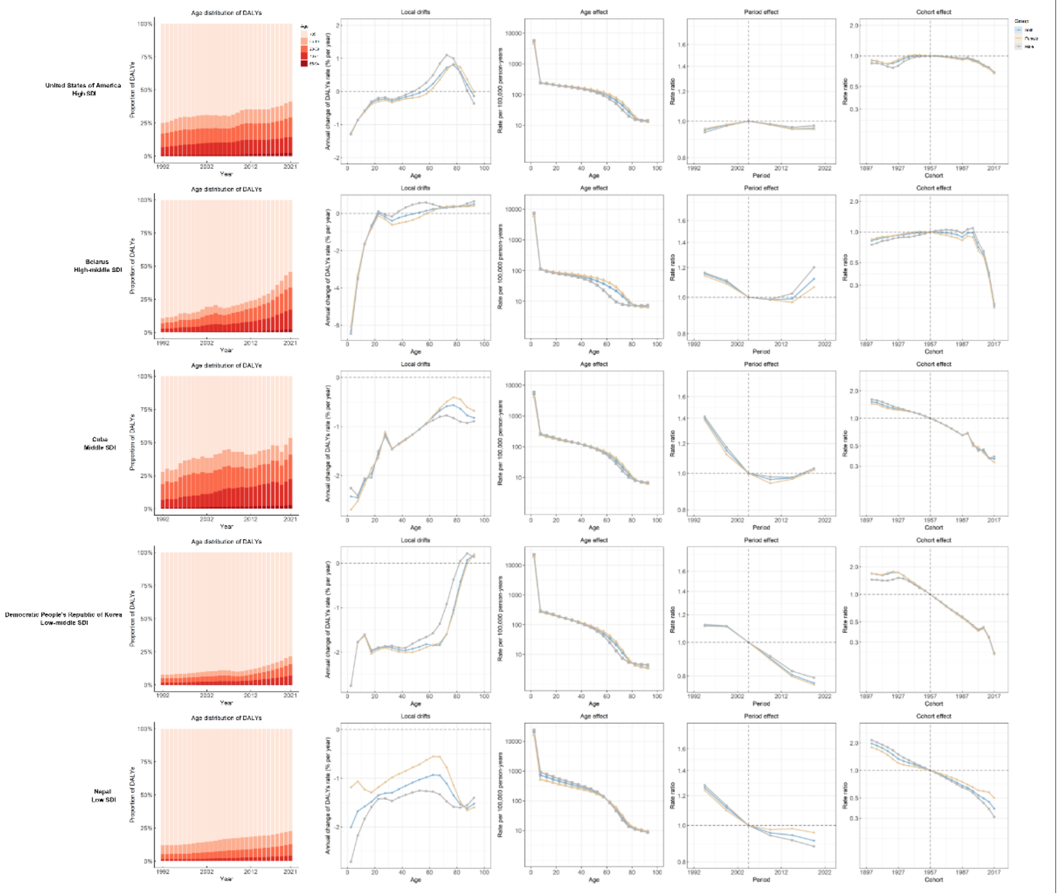

Figure S26 Favorable age-period-cohort effects on exemplar countries across SDI quintiles. Local drifts indicate the annual percentage change of incidence rate (% per year) across five-year age groups (from 0-4 to 90-94 years). Age effects are represented by the fitted longitudinal age curves of disability-adjusted life years rate (per 100,000 person-years) adjusted for period deviations. Period effects are represented by the relative risk of disability-adjusted life years rate (incidence rate ratio) and computed as the ratio of age-specific rates in each period compared to the referent 2002-2006 period. Cohort effects are represented by the relative risk of disability-adjusted life years rate (rate ratio) and computed as the ratio of age-specific rates in each cohort compared to the referent 1957 cohort. The shaded areas indicate the corresponding 95% CIs of each point estimate. SDI=Socio-demographic Index.

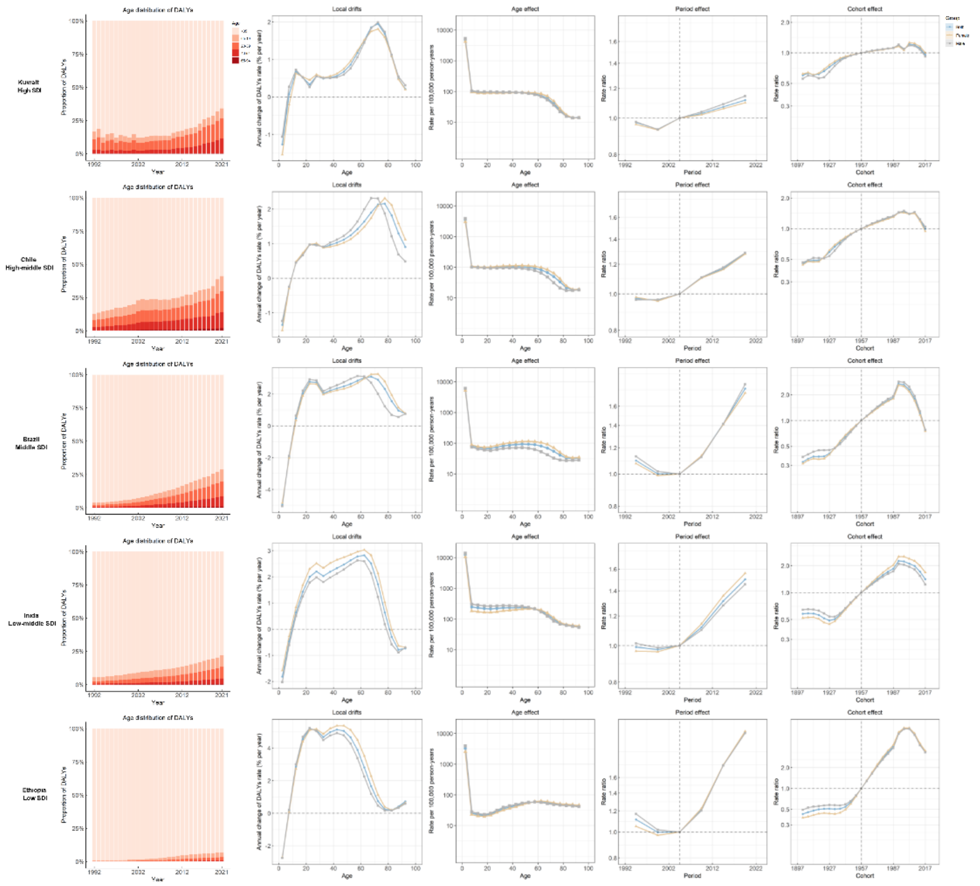

Figure S27. Unfavorable age-period-cohort effects on exemplar countries across SDI quintiles. Local drifts indicate the annual percentage change of incidence rate (% per year) across five-year age groups (from 0-4 to 90-94 years). Age effects are represented by the fitted longitudinal age curves of disability-adjusted life years rate (per 100,000 person-years) adjusted for period deviations. Period effects are represented by the relative risk of disability-adjusted life years rate (incidence rate ratio) and computed as the ratio of age-specific rates in each period compared to the referent 2002-2006 period. Cohort effects are represented by the relative risk of disability-adjusted life years rate (rate ratio) and computed as the ratio of age-specific rates in each cohort compared to the referent 1957 cohort. The shaded areas indicate the corresponding 95% CIs of each point estimate. SDI=Socio-demographic Index.

Table S1 204 countries and territories by sociodemographic index

High SDI (N=40)	High-middle SDI (N=47)	Middle SDI (N=41)	Low-middle SDI (N=43)	Low SDI (N=33)
Andorra	American Samoa	Albania	Angola	Afghanistan
Australia	Antigua and Barbuda	Algeria	Bangladesh	Benin
Austria	Argentina	Armenia	Belize	Burkina Faso
Belgium	Bahamas	Azerbaijan	Bhutan	Burundi
Bermuda	Bahrain	Botswana	Bolivia	Central African Republic
Canada	Barbados	Brazil	Cabo Verde	Chad
Cyprus	Belarus	Colombia	Cambodia	Côte d'Ivoire
Czechia	Bosnia and Herzegovina	Costa Rica	Cameroon	Democratic Republic of the Congo
Denmark	Brunei Darussalam	Cuba	Comoros	Eritrea
Estonia	Bulgaria	Dominican Republic	Congo	Ethiopia
Finland	Chile	Ecuador	Democratic People's Republic of Korea	Gambia
France	China	Equatorial Guinea	Djibouti	Guinea
Germany	Cook Islands	Fiji	Egypt	Guinea-Bissau
Greenland	Croatia	Gabon	El Salvador	Haiti
Iceland	Dominica	Grenada	Eswatini	Liberia
Ireland	Georgia	Guyana	Ghana	Madagascar
Japan	Greece	Indonesia	Guatemala	Malawi

Kuwait	Guam	Iran	Honduras	Mali
Latvia	Hungary	Iraq	India	Mozambique
Lithuania	Israel	Jamaica	Kenya	Nepal
Luxembourg	Italy	Maldives	Kiribati	Niger
Monaco	Jordan	Mexico	Kyrgyzstan	Papua New Guinea
Netherlands	Kazakhstan	Nauru	Lao People's Democratic Republic	Rwanda
New Zealand	Lebanon	Palestine	Lesotho	Senegal
Norway	Libya	Panama	Marshall Islands	Sierra Leone
Poland	Malaysia	Paraguay	Mauritania	Solomon Islands
Puerto Rico	Malta	Peru	Micronesia (Federated States of)	Somalia
Qatar	Mauritius	Philippines	Mongolia	South Sudan
Republic of Korea	Montenegro	Saint Lucia	Morocco	Timor-Leste
San Marino	Niue	Saint Vincent and the Grenadines	Myanmar	Togo
Saudi Arabia	North Macedonia	South Africa	Namibia	Uganda
Singapore	Northern Mariana Islands	Sri Lanka	Nicaragua	United Republic of Tanzania
Slovenia	Oman	Suriname	Nigeria	Yemen
Sweden	Palau	Syrian Arab Republic	Pakistan	
Switzerland	Portugal	Thailand	Samoa	
Taiwan (Province of China)	Republic of Moldova	Tokelau	Sao Tome and Principe	

United Arab Emirates	Romania	Tonga	Sudan
United Kingdom	Russian Federation	Tunisia	Tajikistan
United States of America	Saint Kitts and Nevis	Turkmenistan	Tuvalu
United States Virgin Islands	Serbia	Uzbekistan	Vanuatu
	Seychelles	Viet Nam	Venezuela (Bolivarian Republic of)
	Slovakia		Zambia
	Spain		Zimbabwe
	Trinidad and Tobago		
	Türkiye		
	Ukraine		
	Uruguay		

SDI: sociodemographic index

Table S2 Time trends in neonatal preterm birth disability-adjusted life years rate for both sexes in 204 countries, 1992-2021

SDI		Disability-adjusted life years		All-age disability-adjusted life years rate		Age-standardized disability-adjusted life years rate		Net drift of disability-adjusted life years rate from APC model, % per year
**Quintil**	**Country**	**Number in 2021**	**Percent change 1992-2021, %**	**Rate in 2021**	**Percent change 1992-2021, %**	**Rate in 2021**	**Percent change 1992-2021, %**	
High SDI	Andorra	77.33 (55.35, 101.2)	-47.65	90.35 (64.67, 118.23)	-62.38	124.02 (91.59, 160.11)	-68.41	-0.089 (-2.539, 2.42)
High SDI	Australia	49146.89 (40140.31, 59855.16)	-33.01	190.55 (155.63, 232.06)	-55.05	257.86 (216.33, 308.67)	-51.87	-0.131 (-0.48, 0.218)
High SDI	Austria	16143.54 (13045.98, 19461.61)	-42.8	179.72 (145.24, 216.66)	-50.2	276.94 (231.12, 324.4)	-49.8	0.938 (0.477, 1.401)
High SDI	Belgium	16523.34 (13415.47, 19715.7)	-43.52	144.06 (116.96, 171.9)	-50.6	225.34 (187.1, 261.7)	-49.95	0.466 (-0.101, 1.038)
High SDI	Bermuda	105.57 (78.86, 136.08)	-55.25	166.15 (124.11, 214.16)	-57.55	236.3 (181.18, 300.3)	-51.6	0.204 (-2.168, 2.635)
High SDI	Canada	80946.45 (66870.16, 95576.2)	-8.06	216.03 (178.46, 255.07)	-31.21	341.89 (286.23, 396.95)	-16.28	0.647 (0.485, 0.809)
High SDI	Cyprus	1731.57 (1369.68, 2118.57)	-67.83	127.53 (100.87, 156.03)	-80.88	173.17 (139.82, 209.79)	-78.11	-0.251 (-1.917, 1.443)
High SDI	Czechia	15248.88 (11998.43, 18705.45)	-66.66	143.41 (112.84, 175.92)	-67.72	209.65 (172.39, 250.78)	-71.19	1.105 (0.275, 1.942)
High SDI	Denmark	11174.57 (9397.7, 12985.9)	-21.82	190.96 (160.59, 221.91)	-30.94	305.36 (259.1, 352.82)	-23.88	0.35 (0.097, 0.604)
High SDI	Estonia	1455.33 (1086.85, 1853.47)	-72.16	111.02 (82.91, 141.39)	-67.26	144.96 (112.42, 180.6)	-72.28	-0.534 (-1.12, 0.054)
High SDI	Finland	5676.91 (4524.56, 6896.71)	-57.67	102.54 (81.73, 124.58)	-61.33	164.89 (137.45, 193.07)	-57.47	0.25 (-0.196, 0.698)
High SDI	France	100452.21 (83320.82, 120709.51)	-25.13	151.3 (125.5, 181.81)	-34.41	240.47 (200.3, 284.46)	-28.03	0.14 (-0.02, 0.301)
High SDI	Germany	195990.7 (154506.14, 238811.41)	-23.71	229.57 (180.98, 279.73)	-27.77	350.34 (290.34, 414.61)	-29.69	0.332 (0.166, 0.497)
High SDI	Greenland	288.76 (236.72, 336.81)	-76.78	514.71 (421.95, 600.35)	-77.07	716.63 (585.06, 839.91)	-67.31	0.391 (-4.644, 5.693)
High SDI	Iceland	557.73 (440.81, 706.46)	-38.59	159.17 (125.8, 201.62)	-54.52	193.9 (155.82, 239.77)	-50.47	0.296 (-0.725, 1.329)
High SDI	Ireland	8208.92 (6836.24, 9769.16)	-27.43	166.12 (138.34, 197.7)	-46.95	235.91 (200.74, 276.03)	-43.5	0.394 (-0.039, 0.83)
High SDI	Japan	106021.6 (80701.93, 131594.11)	-38.34	83.02 (63.19, 103.05)	-38.88	127.36 (102.24, 151.63)	-42.33	0.804 (0.361, 1.25)
High SDI	Kuwait	14289.84 (12072.69, 16671.6)	45.18	307.3 (259.62, 358.52)	-47.06	487.19 (411.44, 575.85)	-17.34	0.849 (-0.075, 1.783)
High SDI	Latvia	1708.32 (1417.55, 2027.66)	-80.52	91.33 (75.79, 108.41)	-72.56	155.6 (133.57, 180.11)	-71.48	-0.389 (-0.878, 0.101)
High SDI	Lithuania	3248.59 (2579.5, 3918.85)	-83.38	119.07 (94.54, 143.63)	-77.53	185.19 (154.46, 216.3)	-75.57	0.311 (-0.367, 0.993)
High SDI	Luxembourg	769.26 (613.19, 941.63)	-17.43	119.4 (95.17, 146.15)	-50.17	169.99 (140.72, 202.83)	-48.2	0.082 (-0.845, 1.019)
High SDI	Monaco	37.28 (27.85, 47.48)	-9.07	98.47 (73.57, 125.4)	-25.52	143.62 (109.27, 177.85)	-34.01	0.065 (-2.428, 2.623)
High SDI	Netherlands	28375.72 (23488.05, 33153.67)	-27.75	164.87 (136.47, 192.63)	-36.49	258.64 (222.48, 296.08)	-30.25	0.693 (0.3, 1.088)
High SDI	New Zealand	11934.5 (10338.8, 13497.4)	-1.79	230.86 (199.99, 261.09)	-33.68	349.95 (307.57, 391.9)	-14.32	0.147 (-0.191, 0.486)
High SDI	Norway	6469.21 (4941.52, 7984.22)	-38.6	119.4 (91.2, 147.36)	-51.43	153.8 (122.96, 185.06)	-51.5	-0.366 (-0.613, -0.119)
High SDI	Poland	73228.44 (60329.16, 86114.33)	-73.61	191.5 (157.77, 225.2)	-73.54	337.55 (279.88, 398.98)	-69.05	0.884 (-0.028, 1.806)
High SDI	Puerto Rico	7810.7 (6256.15, 9530.08)	-81.15	237.09 (189.9, 289.28)	-78.96	495.13 (419.7, 581.94)	-61.89	0.393 (-0.289, 1.081)
High SDI	Qatar	9118.64 (7239.24, 11294.95)	54.3	306.29 (243.16, 379.39)	-75.48	368 (295.56, 452.7)	-65.51	0.079 (-3.715, 4.024)
High SDI	Republic of Korea	48891.03 (37386.12, 61268.17)	-74.4	94.8 (72.49, 118.8)	-77.73	182.31 (146.35, 219.7)	-65.5	-0.168 (-0.684, 0.35)
High SDI	San Marino	31.01 (22.17, 40.36)	-35.45	94.74 (67.73, 123.28)	-51.79	129.38 (93.37, 167.9)	-58.31	0.109 (-2.869, 3.18)
High SDI	Saudi Arabia	85100.7 (67490.53, 103677.22)	-84.37	225.7 (179, 274.97)	-92.92	298.96 (234.52, 363.91)	-86.54	0.18 (-2.814, 3.267)
High SDI	Singapore	7555.37 (5784.79, 9706.44)	-7.25	131.91 (101, 169.47)	-47.68	170.54 (137.76, 211.47)	-39.33	0.662 (0.037, 1.29)
High SDI	Slovenia	2514.06 (1995.22, 3050.34)	-61.64	121.47 (96.4, 147.38)	-63.28	187.64 (156.16, 220.52)	-67.7	0.239 (-0.221, 0.701)
High SDI	Sweden	16651.86 (12931.85, 20635.15)	-22.72	160.52 (124.66, 198.92)	-35.17	207.23 (166.88, 250.45)	-36.55	0.832 (0.577, 1.088)
High SDI	Switzerland	13694.66 (11068.28, 16493.6)	-25.54	153.47 (124.04, 184.84)	-41.85	229.98 (193.25, 267.89)	-41.4	0.476 (0.294, 0.658)
High SDI	Taiwan (Province of China)	31862.1 (25743.24, 38452.06)	-34.2	134.79 (108.91, 162.67)	-42.11	249.09 (213.19, 288.49)	-8.78	0.479 (-0.217, 1.181)
High SDI	United Arab Emirates	26089.21 (21033.61, 31624.27)	-12.97	270.88 (218.39, 328.35)	-81.05	421.67 (341.96, 505.53)	-67.23	0.24 (-2.155, 2.696)
High SDI	United Kingdom	150552.07 (129804.59, 172898.84)	-28.56	221.89 (191.31, 254.83)	-39.3	379.61 (328.08, 429.67)	-28.32	0.469 (0.278, 0.659)
High SDI	United States of America	993292.9 (859508.23, 1134509.95)	-28.4	298.6 (258.38, 341.05)	-44.11	461.07 (407.51, 514.33)	-30.9	-0.023 (-0.153, 0.106)
High SDI	United States Virgin Islands	223.54 (176.19, 271.13)	-82.73	260.23 (205.11, 315.63)	-78.49	440.83 (340.82, 540.32)	-62.67	0.587 (-1.752, 2.984)
High-middle SDI	American Samoa	224.05 (180.88, 273.39)	-60.35	450.28 (363.52, 549.43)	-58.87	590.8 (470.52, 727.08)	-18.05	-0.278 (-5.371, 5.088)
High-middle SDI	Antigua and Barbuda	377.75 (329.32, 433.66)	-34.14	422.56 (368.39, 485.1)	-54.16	639.46 (558.39, 726.47)	-31.69	0.074 (-2.648, 2.873)
High-middle SDI	Argentina	193548.87 (160414.11, 231452.32)	-64.08	425.5 (352.65, 508.82)	-73.23	660.04 (542.75, 794.99)	-59.64	0.396 (-0.179, 0.975)
High-middle SDI	Bahamas	1285.01 (1039.49, 1541.81)	-56.14	331.21 (267.93, 397.41)	-69.77	504.24 (415.08, 605.42)	-48.68	0.091 (-1.56, 1.771)
High-middle SDI	Bahrain	4550.6 (3730.21, 5453.32)	-46.64	297.5 (243.86, 356.52)	-81.29	404.74 (340.16, 474.23)	-69.31	0.893 (-3.785, 5.799)
High-middle SDI	Barbados	1050.05 (821.74, 1312.44)	-42.93	351.16 (274.8, 438.9)	-51.61	672.52 (512.06, 866.33)	-26.29	0 (-1.249, 1.266)
High-middle SDI	Belarus	10173.6 (8227.54, 12300.01)	-83.02	109.1 (88.23, 131.9)	-80.9	191.04 (156.38, 228.52)	-79.7	-0.271 (-0.858, 0.318)
High-middle SDI	Bosnia and Herzegovina	8618.73 (7159.65, 10222.53)	-83.23	260.94 (216.77, 309.5)	-77.17	513.16 (422.28, 614.91)	-67.74	-0.219 (-1.081, 0.65)
High-middle SDI	Brunei Darussalam	2117.02 (1721.61, 2545.39)	7.97	469.26 (381.61, 564.21)	-34.43	607.72 (497.94, 730.95)	2.58	0.336 (-1.952, 2.678)
High-middle SDI	Bulgaria	13370.84 (11305.26, 15514.79)	-55.28	197 (166.57, 228.59)	-43.72	387.44 (327.61, 449.97)	-38.53	-0.095 (-0.574, 0.384)
High-middle SDI	Chile	51678.18 (43654.15, 59403.19)	-49.41	274.91 (232.22, 316.01)	-63.12	423.24 (366.99, 483.32)	-40.89	1.18 (0.844, 1.516)
High-middle SDI	China	2113784.02 (1782328.65, 2466912.32)	-80.58	148.57 (125.27, 173.39)	-83.53	301.59 (260.49, 351.77)	-71.54	0.19 (-0.457, 0.841)
High-middle SDI	Cook Islands	39.47 (27.6, 51.75)	-77.72	222.43 (155.52, 291.59)	-75.96	288.6 (197.07, 387.34)	-66.08	0.027 (-6.064, 6.514)

Table 2 (Continued)

SDI		Disability-adjusted life years		All-age disability-adjusted life years rate		Age-standardized disability-adjusted life years rate		Net drift of disability-adjusted life years rate from APC model, % per year
Quintil	Country	Number in 2021	Percent change 1992-2021, %	Rate in 2021	Percent change 1992-2021, %	Rate in 2021	Percent change 1992-2021, %	
High-middle SDI	Croatia	4867.28 (3964.77, 5884.82)	-69.45	115.64 (94.2, 139.82)	-65.07	205.02 (169.07, 246.51)	-64.69	-0.144 (-0.609, 0.323)
High-middle SDI	Dominica	415.41 (311.74, 550.2)	-42.09	619.31 (464.76, 820.25)	-38.06	1153.67 (814.75, 1594.32)	38.6	0.2 (-2.524, 3.001)
High-middle SDI	Georgia	12232.7 (10119.8, 14880.96)	-73.54	339.11 (280.53, 412.52)	-60.09	527.42 (432.16, 647.24)	-58.23	0.099 (-0.437, 0.639)
High-middle SDI	Greece	288.76 (236.72, 336.81)	-76.78	514.71 (421.95, 600.35)	-77.07	716.63 (585.06, 839.91)	-67.31	3.052 (1.979, 4.137)
High-middle SDI	Guam	980.61 (805.67, 1187.12)	-20.93	615.97 (506.08, 745.7)	-29.15	748.67 (612.07, 910.27)	12.62	0.028 (-2.827, 2.968)
High-middle SDI	Hungary	20199.76 (16443.34, 24061.66)	-76.57	210.47 (171.33, 250.71)	-74.69	348.99 (288.54, 412.69)	-75.1	0.599 (-0.116, 1.319)
High-middle SDI	Israel	14798.42 (11872.22, 18129.22)	-62	154.24 (123.74, 188.96)	-79.24	161.44 (130.13, 197.14)	-77.81	0.063 (-0.781, 0.915)
High-middle SDI	Italy	62600.38 (50405.49, 74170.99)	-66.19	104.66 (84.27, 124)	-67.95	189.95 (159.69, 221.49)	-69.45	0.181 (-0.248, 0.613)
High-middle SDI	Jordan	84962.49 (71904.41, 100313.81)	-29.4	689.37 (583.42, 813.93)	-76.95	773.99 (651.6, 919.28)	-58.14	0.622 (-1.323, 2.607)
High-middle SDI	Kazakhstan	110754.16 (93097.37, 129902.99)	-48.27	584.28 (491.13, 685.3)	-55.76	564.56 (475.26, 661.98)	-56.89	-0.596 (-1.492, 0.307)
High-middle SDI	Lebanon	23477.7 (17973.27, 30749.7)	-71.26	423.75 (324.4, 555)	-83.92	564.52 (425.62, 748.03)	-71.93	-0.325 (-1.199, 0.556)
High-middle SDI	Libya	44053.43 (34725.25, 55872.13)	-59.56	641.19 (505.42, 813.2)	-74.51	1073.94 (830.51, 1379.12)	-41.04	-0.507 (-1.362, 0.354)
High-middle SDI	Malaysia	104008.28 (86030.45, 123792.35)	-36.5	326.94 (270.43, 389.13)	-62.53	385.54 (323.1, 454.9)	-43.47	0.023 (-1.2, 1.263)
High-middle SDI	Malta	1141.84 (950.54, 1353.45)	-51.5	258.2 (214.94, 306.05)	-58.61	445.87 (373.11, 532.55)	-48.65	0.648 (-0.299, 1.604)
High-middle SDI	Mauritius	7524.3 (6330.36, 8611.58)	-59.02	591.55 (497.68, 677.03)	-63.87	1003.13 (853.27, 1138.97)	-38.1	1.299 (-0.272, 2.896)
High-middle SDI	Montenegro	1055.53 (807.56, 1302.32)	-74.57	170.78 (130.66, 210.71)	-74.07	235.33 (177.58, 292.63)	-72.39	0.399 (-0.571, 1.379)
High-middle SDI	Niue	23.91 (20.54, 27.49)	3.66	1430.56 (1228.76, 1644.84)	39.33	2099.66 (1794.51, 2419.49)	90.87	0.59 (-14.499, 18.343)
High-middle SDI	North Macedonia	5649.83 (4908.92, 6532.56)	-82.15	259.59 (225.54, 300.14)	-83.64	519.45 (450.16, 599.92)	-73.84	-0.212 (-0.976, 0.557)
High-middle SDI	Northern Mariana Islands	174.92 (145.03, 209.83)	-48.42	360.73 (299.09, 432.71)	-49.18	502.64 (415.18, 599.75)	-11.69	0.04 (-7.084, 7.711)
High-middle SDI	Oman	174.92 (145.03, 209.83)	-48.42	360.73 (299.09, 432.71)	-49.18	502.64 (415.18, 599.75)	-11.69	0.401 (-1.566, 2.409)
High-middle SDI	Palau	102.45 (85.46, 123.36)	-60.98	566.16 (472.28, 681.69)	-65.43	1063.52 (882.56, 1295.57)	-34.67	0.307 (-8.505, 9.969)
High-middle SDI	Portugal	13606.81 (10655.08, 16638.81)	-64.66	128.27 (100.44, 156.85)	-66.18	211.43 (175.86, 249.59)	-66.95	0.828 (0.272, 1.387)
High-middle SDI	Republic of Moldova	5784.06 (4672.68, 7155.67)	-70.42	160.96 (130.03, 199.13)	-63.43	319.97 (257.49, 407.11)	-42.11	-0.079 (-0.522, 0.365)
High-middle SDI	Romania	46049.04 (40448.98, 52100.59)	-61.28	243.14 (213.58, 275.1)	-52.37	440.3 (392.69, 491.41)	-48.09	-0.007 (-0.38, 0.366)
High-middle SDI	Russian Federation	226211.17 (197680.14, 257884.21)	-64.77	156.16 (136.46, 178.03)	-63.09	273.91 (245.84, 304.08)	-64.56	0.861 (0.441, 1.283)
High-middle SDI	Saint Kitts and Nevis	288.42 (235.47, 350.01)	-45.77	491.8 (401.52, 596.83)	-61.47	879.15 (708.09, 1090.65)	-29.4	-0.015 (-3.958, 4.09)
High-middle SDI	Serbia	18394.25 (15663.46, 21360.33)	-83.55	206.22 (175.6, 239.47)	-82.14	427.51 (367.27, 494.51)	-75.14	-0.237 (-1.598, 1.143)
High-middle SDI	Seychelles	595.54 (469.86, 745.35)	1.91	564.86 (445.65, 706.95)	-28.05	728.59 (572.1, 921.58)	-1.96	-0.257 (-2.682, 2.227)
High-middle SDI	Slovakia	13007.41 (10969.7, 15195.55)	-65.34	239.57 (202.04, 279.87)	-66.03	396.52 (336.84, 462.72)	-60.02	-0.468 (-0.764, -0.17)
High-middle SDI	Spain	50862.36 (40926.29, 62527.39)	-49.15	111.66 (89.85, 137.27)	-56.5	192.6 (163.82, 224.42)	-57.62	0.331 (0.083, 0.581)
High-middle SDI	Trinidad and Tobago	8941.98 (7238.01, 10951.39)	-53.14	641.86 (519.54, 786.09)	-58.82	975.05 (783.93, 1215.65)	-43.95	0.574 (-0.183, 1.337)
High-middle SDI	Turkey	372449.17 (307177.53, 447977.4)	-82.73	445.44 (367.38, 535.78)	-87.69	654.91 (533.65, 791.61)	-78.93	-0.468 (-1.496, 0.57)
High-middle SDI	Ukraine	60753.17 (50971.99, 70096.89)	-76.55	141.03 (118.32, 162.72)	-71.43	354.34 (295.99, 413.38)	-59.27	0.72 (0.395, 1.046)
High-middle SDI	Uruguay	7928.44 (6435.4, 9475.63)	-73.31	232.8 (188.96, 278.23)	-75.09	375.38 (306.59, 451.89)	-65.99	0.575 (-0.214, 1.371)
Middle SDI	Albania	8675.93 (6304.9, 11304.66)	-77.27	325.2 (236.32, 423.73)	-71.6	590.85 (416.12, 786.39)	-42.87	-1.449 (-2.005, -0.889)
Middle SDI	Algeria	517723.04 (383819.62, 671722.08)	-50.3	1171.37 (868.41, 1519.8)	-70.09	1180.65 (874.69, 1532.65)	-58.87	0.005 (-0.381, 0.394)
Middle SDI	Armenia	9175.99 (7683.05, 10760.82)	-83.04	306.34 (256.5, 359.25)	-80.59	474.12 (397.27, 558.23)	-71.51	0.348 (-0.544, 1.248)
Middle SDI	Azerbaijan	83011.33 (63513.62, 101062.53)	-31.12	790.57 (604.88, 962.48)	-50.47	1225.92 (923.53, 1496.81)	-13.69	-0.217 (-0.835, 0.404)
Middle SDI	Botswana	35925.64 (26729.58, 47243.03)	-1.72	1501.12 (1116.87, 1974)	-42.28	1525.95 (1131.61, 2011.39)	-7.56	0.567 (-0.43, 1.575)
Middle SDI	Brazil	1025826.75 (863688.48, 1200736.53)	-68.06	465.53 (391.95, 544.9)	-77.78	592.87 (498.38, 701.51)	-71.25	2.112 (0.31, 3.947)
Middle SDI	Colombia	182268.13 (142259.94, 242084.44)	-73.24	371.51 (289.96, 493.44)	-81.48	498.67 (382.35, 676.34)	-67.03	1.402 (0.239, 2.578)
Middle SDI	Costa Rica	15948.42 (13458.09, 18742.66)	-55.03	335.9 (283.45, 394.75)	-69.44	526.51 (444.37, 622.11)	-43.16	0.029 (-0.508, 0.57)
Middle SDI	Cuba	16215.99 (13335.26, 19274.01)	-67.1	143.89 (118.33, 171.02)	-67.89	236.12 (202.26, 271.91)	-59.41	-1.177 (-1.488, -0.865)
Middle SDI	Dominican Republic	172824.35 (139468.84, 213160.02)	-38.38	1569.29 (1266.41, 1935.54)	-58.36	1667.53 (1344.16, 2058.67)	-35.75	0.492 (-0.016, 1.004)
Middle SDI	Ecuador	108121.87 (88225.97, 132366.69)	-40.72	598.5 (488.37, 732.7)	-65.63	680.72 (553.24, 838.07)	-43.91	-0.176 (-0.753, 0.404)
Middle SDI	Egypt	693531.32 (559598.22, 844468.91)	-61.67	656.58 (529.78, 799.47)	-79.13	575.78 (466.17, 697.66)	-73.26	-0.265 (-2.164, 1.67)
Middle SDI	Equatorial Guinea	22309.01 (14310.25, 33746.53)	52.06	1475.15 (946.25, 2231.45)	-53.8	1241.37 (796.4, 1878.95)	-13.91	1.313 (-0.546, 3.207)
Middle SDI	Fiji	8492.89 (6594.26, 10944.65)	6.72	918.67 (713.29, 1183.87)	-11.07	963.18 (745.82, 1243.89)	8.48	0.134 (-1.458, 1.751)
Middle SDI	Gabon	21175.37 (14848.85, 29000.07)	-6.08	1165.99 (817.63, 1596.84)	-46.69	1027.94 (720.72, 1406.68)	-21.87	0.922 (-0.168, 2.025)
Middle SDI	Grenada	518.57 (430.42, 610.05)	-56.14	505.3 (419.4, 594.44)	-62.24	712.78 (588.1, 851.17)	-34.27	0.215 (-2.283, 2.779)
Middle SDI	Guyana	10668.01 (8060.64, 13757.45)	-70.55	1394.98 (1054.03, 1798.97)	-70.01	1467.16 (1107.21, 1894.4)	-51.36	-0.112 (-1.248, 1.036)
Middle SDI	Indonesia	2095085.26 (1635660.37, 2588175.69)	-46.05	751.15 (586.43, 927.94)	-63.16	958.36 (742.84, 1184.88)	-45	1.68 (0.432, 2.944)
Middle SDI	Iran (Islamic Republic of)	221228.53 (175666.95, 264119.84)	-90.43	259.18 (205.8, 309.43)	-93.26	383.69 (302.37, 465.39)	-88.33	1.313 (0.013, 2.629)

Table 2 (Continued)

SDI		Disability-adjusted life years		All-age disability-adjusted life years rate		Age-standardized disability-adjusted life years rate		Net drift of disability-adjusted life years rate from APC model, % per year
Quintil	Country	Number in 2021	Percent change 1992-2021, %	Rate in 2021	Percent change 1992-2021, %	Rate in 2021	Percent change 1992-2021, %	
Middle SDI	Iraq	535322.36 (423419.64, 657892.34)	-50.58	1298.55 (1027.11, 1595.88)	-77.15	1317.74 (1037.83, 1624.7)	-60.78	
Middle SDI	Jamaica	23539.38 (18403.72, 30054.36)	-61.49	840.72 (657.3, 1073.41)	-66.88	1320.89 (1005.78, 1700.83)	-40.53	
Middle SDI	Mexico	592075.91 (495134.59, 698590.87)	-64.85	457.99 (383, 540.38)	-75.84	614.18 (507.59, 731.75)	-55.44	
Middle SDI	Nauru	155.16 (115.04, 209.82)	-17.08	1407.49 (1043.51, 1903.29)	-22.21	1126.26 (839.86, 1515.27)	-1.66	
Middle SDI	Panama	20663.85 (16816.92, 25178.04)	-27.25	481.45 (391.82, 586.62)	-57.76	576.97 (466.12, 703.71)	-42.37	
Middle SDI	Palestine	41878.53 (33593.82, 51952.76)	-56.23	815.45 (654.13, 1011.62)	-81.16	728.03 (585.01, 902.91)	-66.79	-0.252 (-1.495, 1.006)
Middle SDI	Paraguay	48551.64 (36902.7, 63210.29)	-39.88	677.25 (514.76, 881.73)	-64.11	758.44 (569.23, 992.54)	-42.67	-0.264 (-0.784, 0.259)
Middle SDI	Peru	139617.98 (101980.54, 182427.18)	-69.48	384.93 (281.16, 502.95)	-81.09	416.89 (303.95, 547.58)	-72.22	-0.389 (-1.408, 0.64)
Middle SDI	Philippines	992308.29 (824743.2, 1188384.04)	-16.52	876.21 (728.25, 1049.35)	-51.23	896.04 (742.51, 1074.42)	-26.66	0.9 (0.15, 1.657)
Middle SDI	Saint Lucia	1335.15 (1072.72, 1686.68)	-48.29	752.14 (604.31, 950.17)	-59.14	1303.76 (1018.2, 1687.74)	-17.14	0.299 (-1.407, 2.035)
Middle SDI	Saint Vincent and the Grenadines	629.47 (502.91, 780.89)	-71.49	551.8 (440.85, 684.54)	-72.53	886.98 (698.69, 1126.89)	-50.11	0.401 (-2.159, 3.029)
Middle SDI	South Africa	699030.68 (578366.77, 852237.6)	-12.05	1229.51 (1017.27, 1498.98)	-40.18	1452.51 (1194.65, 1777.31)	-9.34	0.921 (-0.01, 1.861)
Middle SDI	Sri Lanka	97196.73 (79217.15, 118302.13)	-53.9	436.44 (355.71, 531.21)	-63.83	554.91 (451.23, 676.34)	-53.6	-0.198 (-0.969, 0.578)
Middle SDI	Suriname	6645.68 (5223.51, 8635)	-40.67	1147.29 (901.77, 1490.72)	-60.08	1500.39 (1166.65, 1969.52)	-43.04	0.06 (-1.136, 1.271)
Middle SDI	Syrian Arab Republic	52573.98 (41270.25, 66079.83)	-84.86	374.71 (294.14, 470.97)	-85.4	482.72 (376.94, 616.96)	-69.82	-0.328 (-2.201, 1.581)
Middle SDI	Thailand	126820.85 (102822.28, 151944.97)	-76.96	190.18 (154.19, 227.86)	-79.85	331.83 (277.89, 384.94)	-69.41	0.171 (-0.999, 1.356)
Middle SDI	Tokelau	17.22 (11.1, 24.95)	17.85	1256.18 (810.28, 1820)	33.4	1931.65 (1221.64, 2839.61)	120.3	0.135 (-19.227, 24.139)
Middle SDI	Tonga	772.65 (596.47, 998.68)	-45.21	726.86 (561.12, 939.5)	-48.82	554.71 (430.17, 713.66)	-40.48	0.059 (-4.296, 4.613)
Middle SDI	Tunisia	61858.59 (49952.31, 74923.49)	-77.49	522.34 (421.8, 632.66)	-83.42	721.3 (579.8, 884.71)	-73.15	-0.202 (-1, 0.602)
Middle SDI	Turkmenistan	45175 (36060.07, 56366.83)	-26.34	875.76 (699.05, 1092.72)	-45.16	852.21 (680.23, 1063.41)	-18.44	0.075 (-0.647, 0.802)
Middle SDI	Uzbekistan	289098.37 (236714.36, 351850.18)	123.35	844.5 (691.48, 1027.81)	42.89	759.99 (622.98, 924.12)	86.88	0.329 (-0.851, 1.524)
Middle SDI	Viet Nam	451098.68 (356783.08, 555959.11)	-68.53	449.89 (355.82, 554.47)	-77.75	567.86 (445.06, 702.13)	-64.92	-0.67 (-1.348, 0.013)
Low-middle SDI	Angola	631060.47 (456925.06, 832325.69)	8.49	1929.46 (1397.04, 2544.83)	-63.45	1119.49 (815.65, 1470.15)	-52.09	1.635 (-1.456, 4.824)
Low-middle SDI	Bangladesh	1893830.38 (1372757.42, 2565374.12)	-71.72	1150.31 (833.81, 1558.2)	-80.47	1313.77 (928.11, 1795.68)	-62.75	-0.238 (-1.525, 1.064)
Low-middle SDI	Belize	3446.56 (2914.41, 4041.26)	-39.16	803.25 (679.23, 941.85)	-72.18	897.46 (758.17, 1057.01)	-53.47	0.238 (-1.656, 2.169)
Low-middle SDI	Bhutan	8852.05 (6368.12, 11692.81)	-73.91	1169.51 (841.34, 1544.82)	-77.81	1425.42 (1016.19, 1893.05)	-57.93	-0.848 (-2.445, 0.775)
Low-middle SDI	Bolivia (Plurinational State of)	92103.81 (69987.35, 116216.67)	-53.74	780.73 (593.25, 985.12)	-73.73	782.61 (593.97, 988.1)	-57.29	0.299 (-0.506, 1.111)
Low-middle SDI	Cabo Verde	3125.35 (2362.69, 4023.4)	-62.85	558.83 (422.46, 719.41)	-75.29	714.72 (528.46, 926.78)	-49.42	0.521 (-0.742, 1.801)
Low-middle SDI	Cambodia	190882.99 (147881.96, 242305.93)	-53.49	1119.93 (867.64, 1421.64)	-70.47	1109.93 (858.79, 1410.2)	-46.59	0.999 (0.23, 1.774)
Low-middle SDI	Cameroon	739146.13 (563613.56, 965556.93)	67.86	2325.84 (1773.5, 3038.28)	-40.9	1505.69 (1152.95, 1961.62)	-20.31	0.836 (0.212, 1.464)
Low-middle SDI	Comoros	13890.76 (9907.55, 19273.77)	-43.35	1866.09 (1330.99, 2589.25)	-63.28	1708.9 (1220.39, 2368.31)	-35.08	0.699 (0.121, 1.28)
Low-middle SDI	Congo	61387.02 (44073.18, 83582.06)	11.27	1138.56 (817.44, 1550.22)	-47.47	1004.47 (721.22, 1367.4)	-18.68	1.587 (0.064, 3.132)
Low-middle SDI	Democratic People's Republic of Korea	77679.58 (57343.96, 101734.32)	-72.66	294.32 (217.27, 385.47)	-78.02	493.72 (356.9, 660.81)	-54.73	-1.708 (-2.005, -1.41)
Low-middle SDI	Djibouti	12598.72 (9163.75, 16607.54)	2.41	1000.94 (728.04, 1319.43)	-59.98	875.08 (636.99, 1152.05)	-39.47	0.952 (-1.395, 3.355)
Low-middle SDI	El Salvador	29248.14 (22798.42, 37558.26)	-77.32	453.44 (353.45, 582.27)	-80.9	501.16 (387.08, 648.82)	-66.9	-0.044 (-0.817, 0.735)
Low-middle SDI	Eswatini	14695.32 (10552.86, 19692.32)	-29.67	1272.06 (913.48, 1704.61)	-47.92	1060.81 (762.13, 1421.24)	-20.71	-0.092 (-2.058, 1.912)
Low-middle SDI	Ghana	351851.17 (228795.21, 527604.85)	0.47	1027.42 (668.09, 1540.63)	-53.93	773.45 (510.63, 1149.71)	-37.23	0.361 (-0.383, 1.111)
Low-middle SDI	Guatemala	111644.65 (88970.58, 138878.42)	-79.44	708.07 (564.27, 880.79)	-88.42	754.37 (601.39, 942.15)	-76.77	-0.209 (-2.767, 2.416)
Low-middle SDI	Honduras	56879.38 (42260.18, 74194.96)	-32.69	562.59 (417.99, 733.86)	-66.8	529.45 (392.64, 691.78)	-48.03	-0.069 (-0.719, 0.585)
Low-middle SDI	India	24143480.54 (19825960.45, 29610574.6)	-37.35	1706.86 (1401.62, 2093.36)	-60.78	2147.44 (1744.35, 2653.89)	-33.16	1.778 (0.655, 2.913)
Low-middle SDI	Kenya	486712.08 (394578.45, 595006.29)	17.33	972.21 (788.17, 1188.52)	-42.28	857.11 (695.07, 1047.37)	-2.48	2.624 (1.207, 4.062)
Low-middle SDI	Kiribati	2548.28 (1958.88, 3209.48)	-27.14	2103.22 (1616.76, 2648.94)	-53.76	1818.99 (1400.25, 2288.04)	-34.29	-0.148 (-4.884, 4.823)
Low-middle SDI	Kyrgyzstan	51076.45 (43662.7, 58861.74)	-23.78	744.22 (636.19, 857.65)	-48.9	682.41 (584.02, 786.27)	-38.71	0.556 (-0.085, 1.203)
Low-middle SDI	Lao People's Democratic Republic	136231.71 (103065.85, 177022.93)	-54.05	1846.57 (1397.01, 2399.48)	-72.64	1623.93 (1230.4, 2110.76)	-53.86	-0.586 (-1.428, 0.262)
Low-middle SDI	Lesotho	39468.71 (29195.26, 52330.5)	-22.84	2105.68 (1557.59, 2791.87)	-35.1	1946.15 (1439.22, 2582.17)	-5.06	-0.654 (-1.572, 0.271)
Low-middle SDI	Maldives	2122.73 (1646.92, 2610.07)	-73.99	410.42 (318.42, 504.64)	-88.12	604.99 (462.31, 758.75)	-70.37	-0.808 (-3.225, 1.668)
Low-middle SDI	Marshall Islands	553.54 (434.58, 705.79)	-39.53	983.67 (772.27, 1254.23)	-49.22	992.58 (775.6, 1272.16)	-23.79	-0.64 (-7.884, 7.173)
Low-middle SDI	Mauritania	83339.68 (63976.36, 105592.71)	0.94	1895.7 (1455.25, 2401.88)	-50.36	1359.56 (1061.82, 1707.79)	-33.78	0.888 (0.27, 1.511)
Low-middle SDI	Micronesia (Federated States of)	696.83 (541.34, 869.59)	-66.45	679.31 (527.73, 847.74)	-65.37	745.47 (573.17, 938.59)	-47.78	-0.209 (-4.093, 3.832)
Low-middle SDI	Mongolia	22026.36 (17191.14, 27739.42)	-48.8	660.2 (515.27, 831.44)	-65.68	602.98 (471.78, 759.18)	-55.04	0.277 (-0.644, 1.208)
Low-middle SDI	Morocco	319590.77 (253539.04, 392358.71)	-72.14	859.65 (681.98, 1055.39)	-80.3	1011.68 (795.91, 1245.55)	-67	-0.05 (-0.96, 0.868)
Low-middle SDI	Myanmar	545285.61 (412255.54, 700906.92)	-37.24	966.48 (730.69, 1242.3)	-53.87	1048.91 (789.94, 1350.05)	-37.74	-0.478 (-0.833, -0.121)
Low-middle SDI	Namibia	30205.2 (22658.36, 39302.1)	-21.22	1242.41 (931.99, 1616.59)	-51.57	1098.41 (823.81, 1428.36)	-28.93	0.458 (-0.568, 1.495)

Table 2 (Continued)

SDI		Disability-adjusted life years		All-age disability-adjusted life years rate		Age-standardized disability-adjusted life years rate		Net drift of disability-adjusted life years rate from APC model, % per year
**Quintil**	**Country**	**Number in 2021**	**Percent change 1992-2021, %**	**Rate in 2021**	**Percent change 1992-2021, %**	**Rate in 2021**	**Percent change 1992-2021, %**	
Low-middle SDI	Nicaragua	43403.46 (33705.14, 55199.72)	-60.49	650.82 (505.4, 827.7)	-75.56	687.36 (528.94, 875.34)	-58.51	-0.315 (-1.133, 0.509)
Low-middle SDI	Nigeria	9030767.79 (6955518.76, 11466421.46)	90.06	3906.62 (3008.88, 4960.26)	-21.46	2328.8 (1793.99, 2950.48)	-1.72	2.544 (1.619, 3.478)
Low-middle SDI	Pakistan	6415496.7 (4957118.47, 8258298.56)	40.02	2723.58 (2104.45, 3505.91)	-31.02	2188.29 (1694.82, 2806.39)	-4.2	1.173 (0.591, 1.759)
Low-middle SDI	Samoa	1877.09 (1403.68, 2451.06)	-33.67	878.62 (657.03, 1147.28)	-47.08	653.72 (491.71, 844.94)	-41.86	-0.401 (-3.397, 2.687)
Low-middle SDI	Sao Tome and Principe	1378.29 (941.08, 1901.32)	-46.97	636.27 (434.44, 877.73)	-69.13	579.43 (394.72, 799.92)	-50.99	1.382 (-1.07, 3.895)
Low-middle SDI	Sudan	1166162.4 (905449.72, 1478695.02)	-43.65	2685.83 (2085.37, 3405.63)	-71.96	2127.04 (1653.03, 2694.99)	-54.87	0.22 (-0.548, 0.995)
Low-middle SDI	Tajikistan	88814.23 (63355.5, 121697.62)	10.98	874.16 (623.58, 1197.81)	-38.91	673.94 (483.01, 917.8)	-20.25	0.188 (-0.241, 0.619)
Low-middle SDI	Tuvalu	113.47 (84.25, 149.5)	-66.33	917.51 (681.27, 1208.86)	-74.06	886.79 (658.27, 1168.43)	-60.04	0.813 (-12.663, 16.37)
Low-middle SDI	Vanuatu	3714.09 (2607.11, 5023.62)	9.63	1186.68 (832.99, 1605.08)	-44.08	905.04 (639.46, 1217.17)	-22.9	0.218 (-3.936, 4.553)
Low-middle SDI	Venezuela (Bolivarian Republic of)	257682.36 (195400.58, 328962.52)	-21.05	967.68 (733.79, 1235.36)	-41.52	1168.08 (878.99, 1494.23)	-8.11	-0.196 (-0.746, 0.356)
Low-middle SDI	Zambia	235225.63 (166651.05, 324430.24)	17.86	1205.34 (853.95, 1662.44)	-49.82	829.46 (591.48, 1137.46)	-23.73	1.24 (-0.055, 2.552)
Low-middle SDI	Zimbabwe	379445.56 (291487.75, 494325.76)	52.26	2433.06 (1869.06, 3169.69)	5.81	1712.02 (1319.92, 2229.82)	26.53	0.525 (-0.427, 1.488)
Low SDI	Afghanistan	301726.47 (224403.73, 404436.9)	10.02	966.38 (718.73, 1295.35)	-63.52	573.89 (433.1, 753.64)	-55.46	-1.239 (-2.073, -0.398)
Low SDI	Benin	457438.75 (350348.8, 594126.33)	61.02	3388.64 (2595.33, 4401.2)	-38.07	1868.96 (1439.56, 2407.55)	-23.64	0.662 (0.209, 1.117)
Low SDI	Burkina Faso	633066.96 (470974.82, 845318.28)	95.79	2781.35 (2069.2, 3713.86)	-13.98	1419.08 (1061.89, 1881.17)	-1.17	-0.743 (-1.21, -0.274)
Low SDI	Burundi	224763.65 (158795.89, 311498.09)	2.52	1700.14 (1201.15, 2356.21)	-54.91	1028.15 (729.47, 1413.89)	-41.25	1.308 (0.377, 2.247)
Low SDI	Central African Republic	154065.08 (111416.94, 207212.16)	29.68	2809.28 (2031.62, 3778.39)	-31.97	1722.41 (1255.13, 2314.66)	-10.08	-1.671 (-1.965, -1.376)
Low SDI	Chad	703616.45 (524990.9, 929832.43)	116.7	3964.17 (2957.8, 5238.68)	-21.78	1756.77 (1314.84, 2309.95)	-16.36	0.326 (-0.678, 1.34)
Low SDI	Côte d'Ivoire	851562.77 (642730.01, 1107307.38)	36.04	3056.37 (2306.84, 3974.27)	-35.77	1913.99 (1451.22, 2479.86)	-18.07	1.302 (-0.29, 2.921)
Low SDI	Democratic Republic of the Congo	1268248.05 (909602.06, 1726795.43)	34.73	1408.88 (1010.46, 1918.28)	-39.67	942.79 (680.41, 1278.02)	-12.27	1.546 (0.741, 2.357)
Low SDI	Eritrea	87358.97 (59742.67, 120642.78)	8.04	1324.04 (905.47, 1828.5)	-48.3	960.74 (666.15, 1321.52)	-29.41	1.044 (-0.367, 2.476)
Low SDI	Ethiopia	2095743.2 (1604998.37, 2755683.98)	-24.9	1923.79 (1473.31, 2529.58)	-62.17	1295.17 (992.35, 1692.66)	-43.54	3.307 (-0.353, 7.101)
Low SDI	Gambia	53766.59 (36702.34, 70311.71)	-6.8	2245.74 (1532.99, 2936.8)	-58.66	1513.58 (1046.71, 1966.47)	-42.94	1.072 (-0.928, 3.114)
Low SDI	Guinea	333518.82 (240552.92, 453368.77)	3.28	2483.43 (1791.19, 3375.85)	-50.04	1428.17 (1036.3, 1933.31)	-34.97	1.25 (0.332, 2.175)
Low SDI	Guinea-Bissau	57050.64 (40303.94, 75417.68)	-10.03	2764.2 (1952.79, 3654.11)	-54.15	1695.19 (1207.09, 2239.47)	-41.54	1.142 (-0.718, 3.039)
Low SDI	Haiti	170561.04 (118448.45, 233649.44)	31.96	1325.87 (920.77, 1816.3)	-31.17	1060.82 (741.44, 1446.9)	-7.71	-1.38 (-1.815, -0.942)
Low SDI	Liberia	125820.33 (89635.75, 175093.25)	-8.29	2304.62 (1641.83, 3207.14)	-66.45	1638.46 (1175.87, 2270.84)	-53.1	1.445 (-0.481, 3.41)
Low SDI	Madagascar	553677.87 (419853.9, 725161.14)	25.73	1938.82 (1470.2, 2539.3)	-44.69	1390.35 (1058.58, 1811.89)	-18.78	1.726 (0.419, 3.05)
Low SDI	Malawi	257510.34 (185359.3, 335825.32)	-40.53	1324.1 (953.1, 1726.8)	-67.93	959.41 (693.02, 1246.76)	-45	1.143 (-0.657, 2.976)
Low SDI	Mali	1818964.53 (1464298.44, 2231140.13)	100.55	7546.27 (6074.88, 9256.24)	-25.08	3640.04 (2940.35, 4462.02)	-15.15	0.727 (0.15, 1.306)
Low SDI	Mozambique	704832.24 (473234.71, 1032402.3)	-3.38	2268.33 (1522.99, 3322.54)	-57.81	1361.17 (927.15, 1979.86)	-46.32	0.64 (-1.024, 2.333)
Low SDI	Nepal	298614.24 (218541.26, 402639.31)	-46.81	959.25 (702.03, 1293.42)	-65.33	955.16 (694.16, 1291.52)	-41.15	-1.232 (-1.435, -1.028)
Low SDI	Niger	825874.48 (618271.24, 1074295.44)	47.85	3298.45 (2469.31, 4290.62)	-49.51	1511.07 (1143.96, 1951.49)	-42.4	1.043 (-0.869, 2.994)
Low SDI	Papua New Guinea	186206.39 (144243.47, 234526.87)	113.81	1779.9 (1378.79, 2241.79)	-11.39	1178.14 (919.45, 1480.15)	-3.49	0.154 (-0.462, 0.774)
Low SDI	Rwanda	146820.32 (100032.89, 199637.12)	-54.4	1106.4 (753.82, 1504.42)	-74.12	841.12 (576.25, 1137.81)	-59.92	1.094 (-0.762, 2.985)
Low SDI	Senegal	292044.57 (201319.13, 383010.54)	-12.05	1841.44 (1269.38, 2415.01)	-55.3	1301.42 (903.97, 1699.8)	-35.94	2.123 (0.542, 3.728)
Low SDI	Sierra Leone	417534.84 (316297.52, 534281.71)	-7.69	4708.72 (3567.02, 6025.32)	-56.61	2936.61 (2223.77, 3761.35)	-41.55	-0.092 (-0.648, 0.466)
Low SDI	Solomon Islands	2777.4 (1958.53, 3742.65)	-0.7	406.31 (286.51, 547.52)	-47.62	308.83 (224.02, 410.01)	-33.38	-0.951 (-3.552, 1.719)
Low SDI	Somalia	542106.02 (333360.73, 775599.63)	108.75	2508.99 (1542.87, 3589.65)	-25.13	1219.91 (763.71, 1738.52)	-21.04	0.494 (-0.172, 1.164)
Low SDI	South Sudan	379584.54 (255046.19, 562042.09)	54.32	3924.35 (2636.81, 5810.7)	-2.43	2120.13 (1438.4, 3125.48)	7.92	0.084 (-0.346, 0.518)
Low SDI	Timor-Leste	23681.37 (18696.39, 29353.46)	-38.24	1694.26 (1337.62, 2100.07)	-64.01	1242.53 (987.72, 1533.31)	-47.92	-1.137 (-2.173, -0.089)
Low SDI	Togo	156651.83 (109763.72, 207789.38)	-2.9	1871.37 (1311.24, 2482.27)	-55.08	1364.36 (961.23, 1803.03)	-34.36	0.649 (-0.234, 1.541)
Low SDI	Uganda	842506.34 (623918.56, 1150365.95)	17.92	1945.06 (1440.41, 2655.81)	-49.84	1143.24 (856.07, 1557.38)	-27.37	1.603 (0.55, 2.668)
Low SDI	United Republic of Tanzania	1040952.78 (692702.33, 1509387.39)	29.25	1780.99 (1185.16, 2582.45)	-39.41	1181.28 (792.73, 1697.21)	-13.74	1.6 (0.81, 2.395)
Low SDI	Yemen	1113659.35 (903320.31, 1390547.03)	-24.56	3310.17 (2684.97, 4133.18)	-67.27	2427.32 (1984.11, 3016.16)	-48.08	-0.239 (-1.462, 0.999)

Notes:

All-age disability-adjusted life years rate: crude disability-adjusted life years rate.

Age-standardized disability-adjusted life years rate is computed by direct standardization with global standard population in GBD 2021.

Net drifts are estimates derived from the age-period-cohort model and denotes overall annual percentage change in disability-adjusted life years rate, which captures the contribution of the effects from calendar time and successive birth cohorts.

Parentheses for all GBD health estimate indicate 95% uncertainty intervals; parentheses for net drift indicate 95% confidence intervals.

SDI: Socio-demographic Index; APC: age-period-cohort.
